# Characterization of essential eggshell proteins from *Aedes aegypti* mosquitoes

**DOI:** 10.1186/s12915-023-01721-z

**Published:** 2023-10-13

**Authors:** Jun Isoe, Carter J. Simington, Max E. Oscherwitz, Alyssa J. Peterson, Alberto A. Rascón, Brooke B. Massani, Roger L. Miesfeld, Michael A. Riehle

**Affiliations:** 1 Department of Entomology, The University of Arizona, Tucson, AZ 85721 USA; 2 Department of Chemistry and Biochemistry, The University of Arizona, Tucson, AZ 85721 USA; 3 Present address: Department of Molecular and Cellular Biology, University of California, Berkeley, Berkeley, CA94720 USA; 4 Present address: Heersink School of Medicine, University of Alabama, Birmingham, AL 35233 USA; 5 Department of Chemistry, San José State University, San José, CA 95192 USA; 6 Present address: School of Molecular Sciences, Arizona State University, Tempe, AZ 85281 USA

**Keywords:** Mosquito, Eggshell, RNA interference, Melanization, Reproduction

## Abstract

**Background:**

Up to 40% of the world population live in areas where mosquitoes capable of transmitting the dengue virus, including *Aedes aegypti*, coexist with humans. Understanding how mosquito egg development and oviposition are regulated at the molecular level may provide new insights into novel mosquito control strategies. Previously, we identified a protein named eggshell organizing factor 1 (EOF1) that when knocked down with RNA interference (RNAi) resulted in non-melanized and fragile eggs that did not contain viable embryos.

**Results:**

In this current study, we performed a comprehensive RNAi screen of putative *A. aegypti* eggshell proteins to identify additional proteins that interact with intracellular EOF1. We identified several proteins essential for eggshell formation in *A. aegypti* and characterized their phenotypes through a combination of molecular and biochemical approaches. We found that Nasrat, Closca, and Polehole structural proteins, together with the Nudel serine protease, are indispensable for eggshell melanization and egg viability. While all four proteins are predominantly expressed in ovaries of adult females, Nudel messenger RNA (mRNA) expression is highly upregulated in response to blood feeding. Furthermore, we identified four additional secreted eggshell enzymes that regulated mosquito eggshell formation and melanization. These enzymes included three dopachrome-converting enzymes (DCEs) and one cysteine protease. All eight of these eggshell proteins were essential for proper eggshell formation. Interestingly, their eggshell surface topologies in response to RNAi did not phenocopy the effect of RNAi-EOF1, suggesting that additional mechanisms may influence how EOF1 regulates eggshell formation and melanization.

**Conclusions:**

While our studies did not identify a definitive regulator of EOF1, we did identify eight additional proteins involved in mosquito eggshell formation that may be leveraged for future control strategies.

**Supplementary Information:**

The online version contains supplementary material available at 10.1186/s12915-023-01721-z.

## Background

Mosquito-borne diseases such as dengue, Zika, yellow fever, and chikungunya are transmitted to humans via *Aedes aegypti* mosquitoes, which coexist with people in many areas of the world. Current control measures are largely insufficient, and resistance is developing against many mosquitocides [1–4]. Thus, there is an ongoing need to develop novel insecticides that can target a range of mosquito physiological stages and processes. It is imperative that control agents specifically target mosquito-selective biochemical processes to decrease potential negative off-target effects. In particular, vertebrates and beneficial arthropods must not be inadvertently harmed by these new control strategies.

Once an *A. aegypti* female mosquito acquires a blood meal early oogenesis is initiated. Mosquitoes possess ~ 100 ovarioles in each of the paired ovaries, which are composed of primary and secondary follicles along with a germarium [5, 6]. Mosquito ovarian follicles develop synchronously throughout oogenesis [7–13], and the spherical follicle gradually transforms into an ellipsoid form as it accumulates vitellogenin yolk proteins. A single layer of follicular epithelial cells surrounding the oocyte is responsible for ecdysteroid production during the early stages of the reproductive cycle and for secreting a majority of eggshell structural components between 18- and 54-h post blood meal (PBM) [5, 6]. Importantly, the timing of biosynthesis, secretion, and formation of eggshell components during oocyte maturation is crucial for completing embryonic development after oviposition.

Due to environmental and external factors, newly deposited mosquito eggs are sensitive to desiccation, which may impact the reproductive success of a species. To combat this issue, when mosquito eggs are laid the vitelline envelope protein-containing the endochorion layer rapidly undergoes maturation via tanning and hardening processes catalyzed by enzymes. In addition, the mosquito forms an embryo-derived extracellular serosal cuticle that protects the developing embryo and larva from desiccation [14–16]. *A. aegypti* eggshell proteins were described more than 25 years ago [17, 18], and several key eggshell enzymes involved in eggshell melanization and protein cross-linking have been characterized [19–27]. Moreover, proteomics studies have been performed on mosquito eggshells to identify the most abundant protein components [6, 28]. However, these descriptive studies have not elucidated the biochemical events that orchestrate eggshell synthesis, nor the molecular identity of key regulatory proteins required for eggshell melanization. Understanding blood meal digestion and reproductive processes in insect vectors of human disease may lead to the development of selective and safe small molecular inhibitors that act to reduce the rate of disease transmission. We previously investigated the biochemical processes required for blood meal digestion in the midgut lumen and for complete synthesis of the mosquito eggshell and embryo viability [29–34] to further investigate this topic, and we discovered that acetyl CoA carboxylase (ACC) and mosquito lineage-specific eggshell organizing factor 1 (EOF1) are essential for complete eggshell formation and melanization in *A. aegypti* [30, 34]. Nearly 100% of eggs oviposited by ACC- and EOF1-deficient females had defective eggshells and non-viable egg phenotypes. However, detailed insights into the molecular mechanisms involved in mosquito eggshell formation and melanization remain poorly understood.

In this study, we identified several additional essential eggshell proteins through an RNAi screen and determined the impact their knockdown had on fecundity, eggshell melanization, and embryo viability. Four proteins, Nasrat, Closca, Polehole, and Nudel, were predominantly expressed in the ovaries of adult females. Furthermore, these proteins were found to be responsible for maintaining eggshell viability and the melanization process without affecting exochorionic ultrastructures. Our eggshell proteomics identified an additional 168 eggshell structural proteins and enzymes, suggesting that mosquito extracellular eggshells are composed of a complex mixture of proteins. These proteins function together to perform the cross-linking, melanization, and sclerotization processes to protect the embryo and larva from the environment for extended periods of time. These data provide new insights into mosquito ovarian maturation and eggshell synthesis that could lead to key advances in the field of vector control.

## Results

### Systematic RNAi screening of mosquito eggshell proteins

**Fig. 1:**
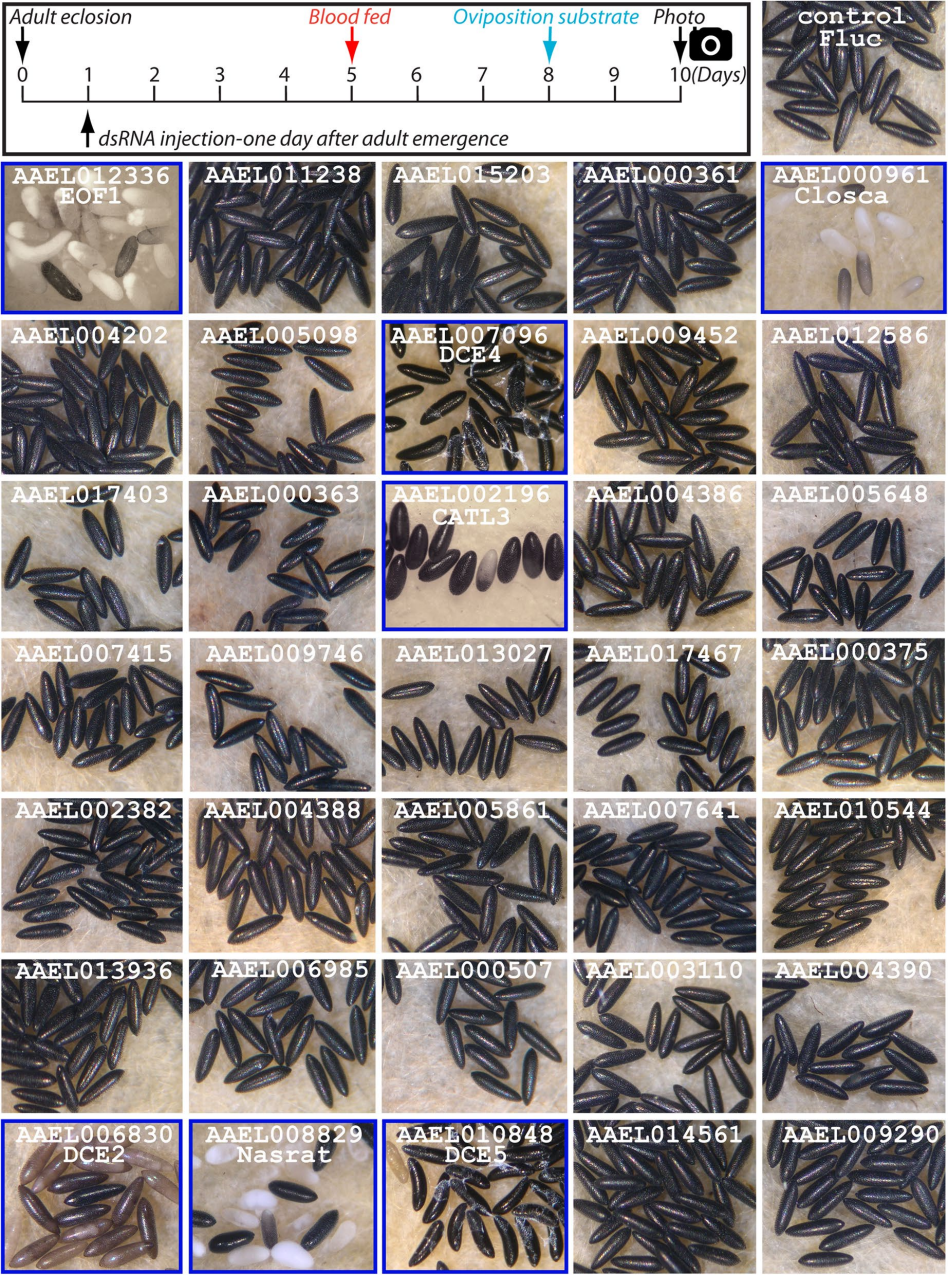
A search for novel essential eggshell proteins in *Aedes aegypti* mosquitoes. Schematic diagram of the experimental time course for dsRNA microinjection, blood feeding, and oviposition in the first gonotrophic cycle shown in the top left corner. Target eggshell proteins were chosen from a proteomics analysis by Marinotti et al. [6]. Representative egg phenotypes associated with RNAi screening of 34 eggshell genes are shown. RNAi against AAEL000961, AAEL002196, AAEL006830, AAEL007096, AAEL008829, and AAEL010848 (as indicated by blue frames) resulted in defective eggshell phenotypes. Eggs from RNAi-firefly luciferase (Fluc) and RNAi-EOF1 (AAEL012336) are also included. Eggs deposited by ten to twenty RNAi mosquitoes for each respective eggshell protein RNAi knockdown were examined under a light microscope and photographed 2 days after oviposition. Putative functions and RNAi primers used for each gene are shown in Additional file 1: Table S1 and Additional file 2: Table S2, respectively

We performed systematic RNAi screen of putative eggshell proteins identified in a previous *A. aegypti* proteomics study [6] to identify downstream targets of EOF1. Predicted functions and RNAi primer sets used for each assayed eggshell protein are shown in Additional file 1: Table S1 and Additional file 2: Table S2, respectively. RNAi screening of 34 eggshell proteins identified six that are essential for proper eggshell formation in oviposited eggs (Fig. 1). These include Nasrat (AAEL008829) and Closca (AAEL000961), which are structural proteins that associate with two other proteins, Polehole and Nudel serine protease, in the perivitelline space (between the eggshell and oocyte plasma membrane) of developing ovarian follicles in *Drosophila melanogaster* [35, 36]. VectorBase BLAST analysis showed that both Nasrat and Closca shared only ~ 25% sequence identity between *A. aegypti* and *D. melanogaster*. Three other RNAi positives, DCE2 (AAEL006830), DCE4 (AAEL007096), and DCE5 (AAEL010848), belong to the dopachrome-converting enzyme family. Cysteine protease cathepsin L-like (CATL3, AAEL002196) was also found to be essential for proper eggshell formation and melanization in *A. aegypti* mosquitoes (Fig. 1). RNAi knockdown of genes encoding the other 28 eggshell proteins showed no observable egg phenotypes, reduced fecundity, or viability. Thus, we focused our effort on these six genes for further characterization.

### Nasrat, Closca, Polehole and Nudel are important for eggshell melanization

**Fig. 2:**
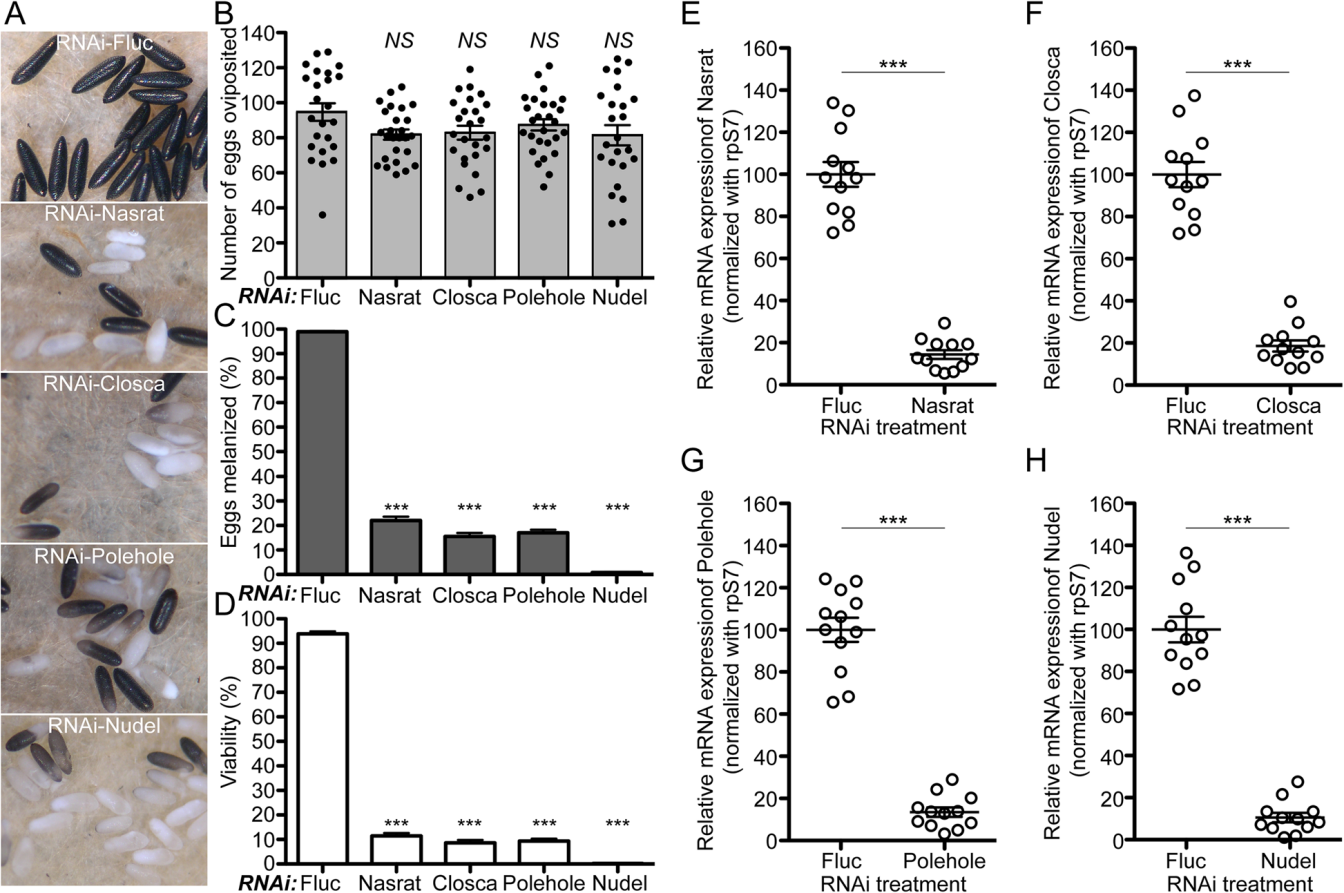
Reproductive phenotypes associated with RNAi knockdown of Nasrat, Closca, Polehole, and Nudel in *Aedes aegypti* mosquitoes. **A** Representative eggs are shown from mosquitoes microinjected with dsRNA against Fluc (control), Nasrat, Closca, Polehole, and Nudel. **B** The effect of RNAi knockdown on fecundity was studied during the first gonotrophic cycle. Each dot represents the number of eggs oviposited by an individual mosquito. **C** Melanization of these eggs was examined under a light microscope and a melanization percentage was determined. **D** An effect of RNAi knockdown on egg viability was examined by hatching eggs 1 week after oviposition. Each bar corresponds to egg viability from 24 to 27 individual mosquitoes from three independent cohorts. Vectorbase ID: Nasrat (AAEL008829), Closca (AAEL000961), Polehole (AAEL022628), and Nudel (AAEL016971). The mean ± SEM are shown as horizontal lines. Statistical significance is represented by stars above each column (unpaired Student’s *t* test; *** *P* < 0.001, *NS* not significant). A detailed phenotypic analysis can be found in Additional file 4: Table S3. Primers used are shown in Additional file 5: Table S4. RNAi-mediated knockdown efficiency was validated by quantitative real-time PCR (qPCR). Relative abundance of mRNA levels for Nasrat (**E**), Closca (**F**), Polehole (**G**), and Nudel (**H**) was analyzed in dissected mosquito ovaries at 36 h PBM. Mosquitoes were microinjected with each dsRNA at 4 days prior to blood feeding, as shown in Fig. 1. dsRNA-Fluc-injected mosquitoes were used as controls. A single mosquito analysis was performed to isolate total RNA, synthesize cDNA, and monitor silencing efficiency by qPCR. mRNA levels were normalized according to transcript levels of ribosomal S7 protein. Data are presented as MEAN ± SEM of 12 individual mosquitoes. *** *P* < 0.001 compared to RNAi-Fluc

Nasrat, Closca, and Polehole form a heterotrimeric protein complex that interacts with the Nudel protease during *D. melanogaster* eggshell formation [35, 36]. Polehole and Nudel are highly diverged proteins, sharing 22 and 41% amino acid sequence identity, respectively, between mosquitoes and fruit flies. Although Polehole and Nudel were not identified as eggshell proteins in previous mosquito proteomics studies [6, 28], we hypothesized that both proteins may play important roles in eggshell formation and melanization in *A. aegypti*. Thus, we performed RNAi-mediated knockdown studies and observed melanization defective egg phenotypes associated with reduced Nasrat, Closca, Polehole, and Nudel expression (Fig. 2A). Melanization processes of eggs deposited by different double-stranded RNA (dsRNA) microinjected female mosquitoes were shown by time-lapse videos (Additional file 3: Video S1). We further characterized reproductive phenotypes, including fecundity, egg melanization, and viability, in the first gonotrophic cycle. As shown in Fig. 2B, there are no significant differences in the number of eggs oviposited among different RNA deficient mosquitoes compared to the firefly luciferase (Fluc) RNAi control (Additional file 4: Table S3), suggesting that these four proteins play no significant roles in blood meal digestion, yolk protein synthesis, or yolk uptake by primary follicles. Conversely, RNAi silencing of these genes had a significant impact on egg melanization and viability. We observed that nearly 99% of eggs laid by mosquitoes in the control group were melanized. Comparatively, 79 to 84% of eggs deposited by Nasrat, Closca, and Polehole deficient mosquitoes did not undergo complete melanization (Fig. 2C, Additional file 4: Table S3). Females exposed to RNAi-Nudel exhibited the most adverse effect on eggshell melanization, with only 1% of their eggshells completing the melanization process. Additionally, nearly 100% of the eggs deposited by Nudel deficient mosquitoes failed to produce viable larvae and immediately deflated when they dried (Fig. 2D, Additional file 4: Table S3). RNAi against Nasrat, Closca, and Polehole resulted in less than 11% egg viability (Fig. 2D, Additional file 4: Table S3). Quantitative real-time PCR (qPCR) using a single mosquito analysis with gene-specific primers (Additional file 5: Table S4) confirmed that the reproductive phenotypes associated with RNAi against *Nasrat*, *Closca*, *Polehole*, and *Nudel* are likely due to the reduced levels of the corresponding endogenous mRNA in the ovaries (Fig. 2E–H).

**Fig. 3:**
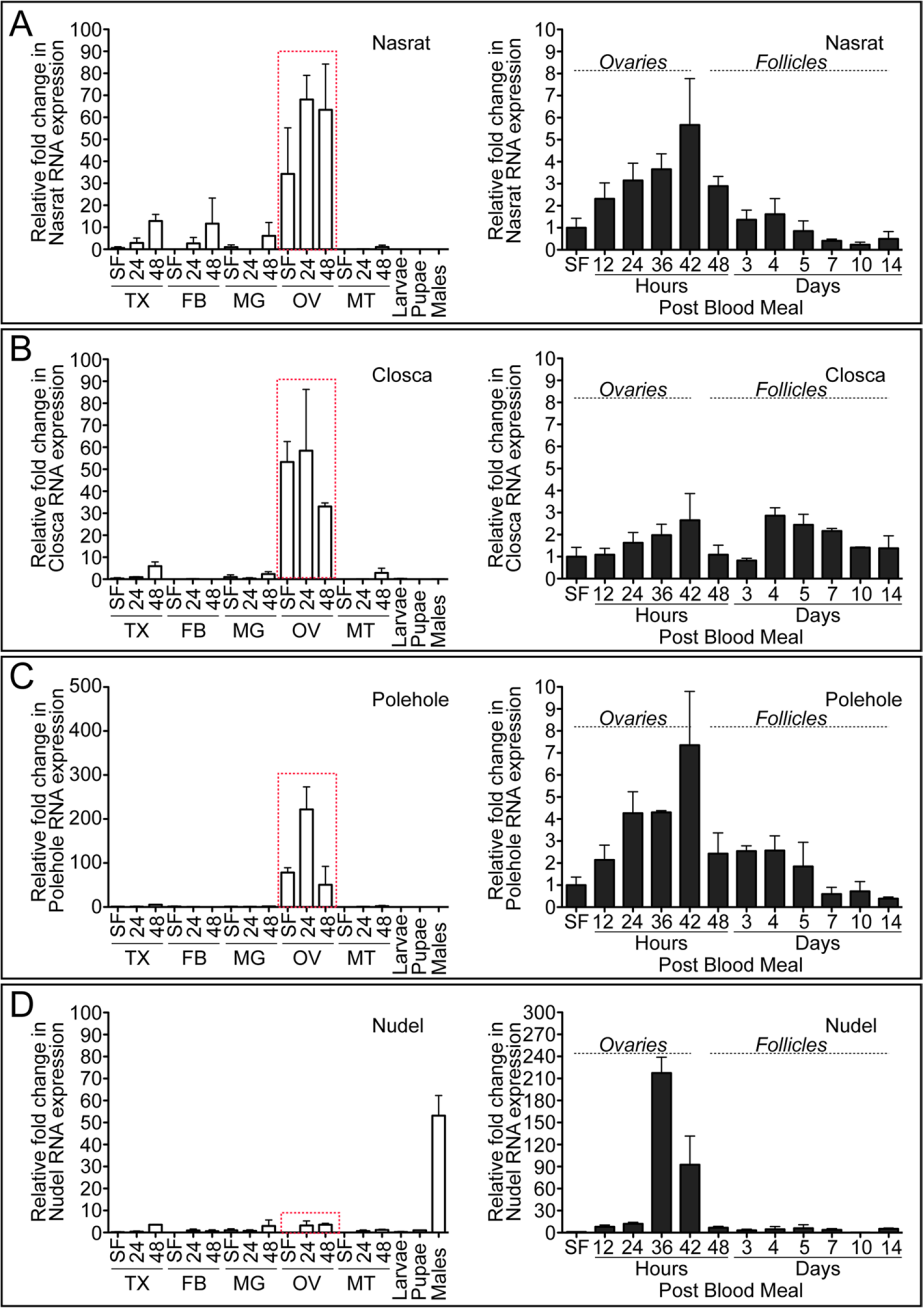
Relative expression of Nasrat, Closca, Polehole, and Nudel during the first gonotrophic cycle in *Aedes aegypti* mosquitoes. Tissue-specific and developmental expression patterns of Nasrat (**A**), Closca (**B**), Polehole (**C**), and Nudel (**D**) during the first gonotrophic cycle are shown in the left-hand graphs. Transcript expression was analyzed by qPCR using cDNAs prepared from thorax (TX), fat body (FB), midgut (MG), ovaries (OV), and Malpighian tubules (MT) in sugar-fed only (SF) or 24 and 48 h PBM. Expression levels were also determined in larvae (Lv), pupae (Pp), and adult males (M). Expression in fat body of SF mosquitoes was set to 1.0. Nasrat, Closca, and Polehole are predominantly expressed in ovaries (highlighted with a red dotted rectangle). Right-hand graphs show more detailed analysis in mosquito ovaries during and after the first gonotrophic cycle was also analyzed by qPCR. Samples from SF to 36 h PBM include entire ovaries, whereas those from 48 h to 14 days PBM include only primary follicles isolated from ovaries. Expression in SF ovaries was set to 1.0 and expression levels for Nasrat, Closca, Polehole, and Nudel were normalized to S7 ribosomal protein transcript levels in the same cDNA samples. Data were collected from three unique mosquito cohorts. The mean ± SEM are shown as horizontal lines

**Fig. 4:**
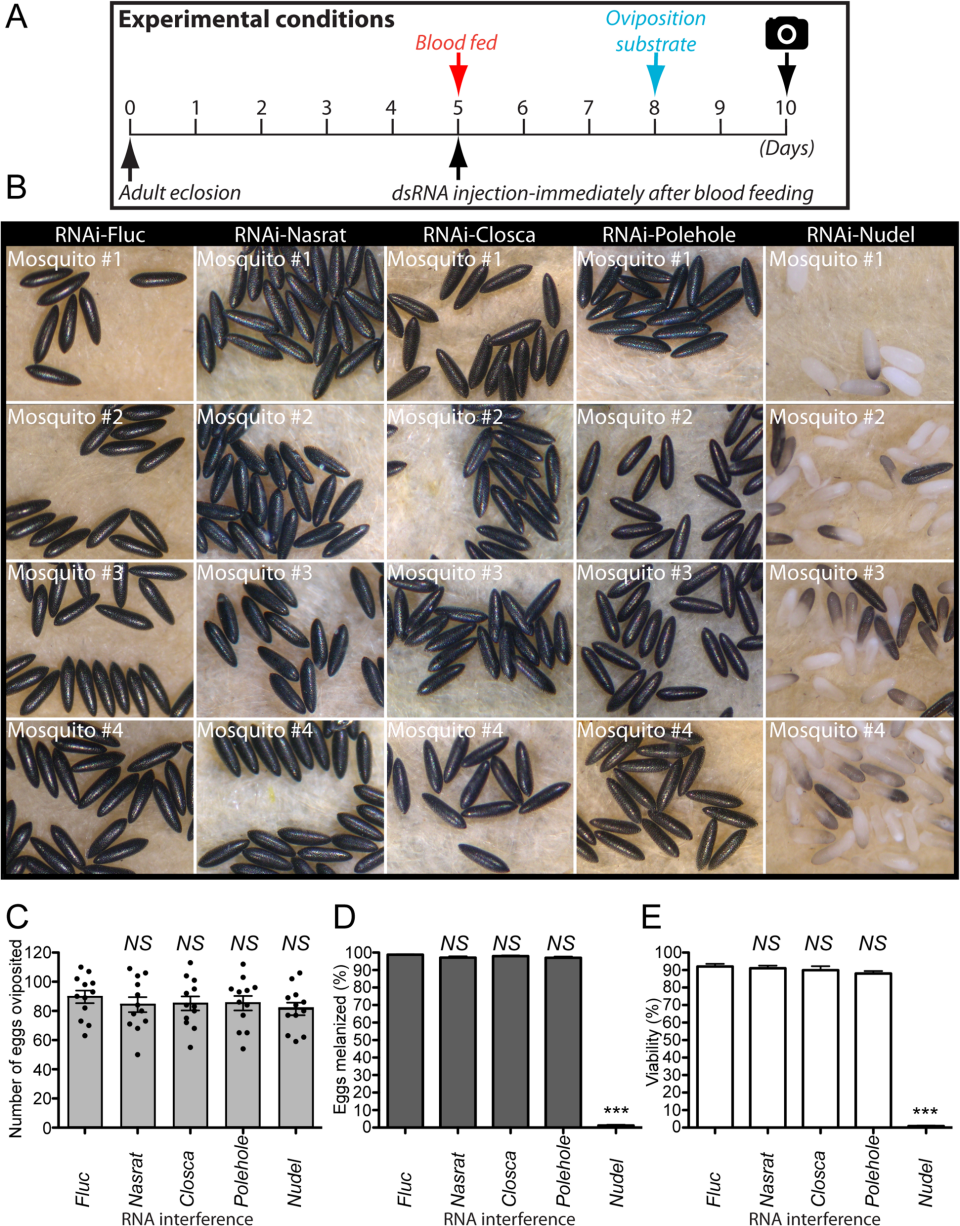
Reproductive phenotypes associated with selected RNAi in response to dsRNA injection immediately after blood feeding. **A** Schematic diagram of experimental time course for dsRNA microinjection, blood feeding, and oviposition in the first gonotrophic cycles. Mosquitoes were microinjected with dsRNA immediately after blood feeding. **B** Representative eggs shown are from four different mosquitoes microinjected with either dsRNA-Fluc, -Nasrat, -Closca, -Polehole, or -Nudel. Female mosquitoes microinjected with dsRNA-Nasrat, -Closca, or -Polehole showed no difference in egg production (**C**), melanization (**D**), and embryo viability (**E**) compared to RNAi-Fluc control mosquitoes. Females microinjected with dsRNA-Nudel immediately after blood feeding produced the similar number of eggs as controls, but had greatly reduced eggshell melanization and viability. The effect of selected RNAi on *A. aegypti* egg production was examined by counting the number of eggs laid by each individual female. Each dot represents the number of eggs oviposited by an individual mosquito (**C**). Egg melanization was examined under a light microscope (**D**) and viability was determined by hatching them 1 week after oviposition (**E**). Each bar corresponds to egg viability from 12 individual mosquitoes from three groups. The mean ± SEM are shown as horizontal lines. Statistical significance is represented by stars above each column (unpaired Student’s *t* test; *** *P* < 0.001, *NS* not significant). A detailed phenotypic analysis is shown in Additional file 6: Table S5

**Fig. 5:**
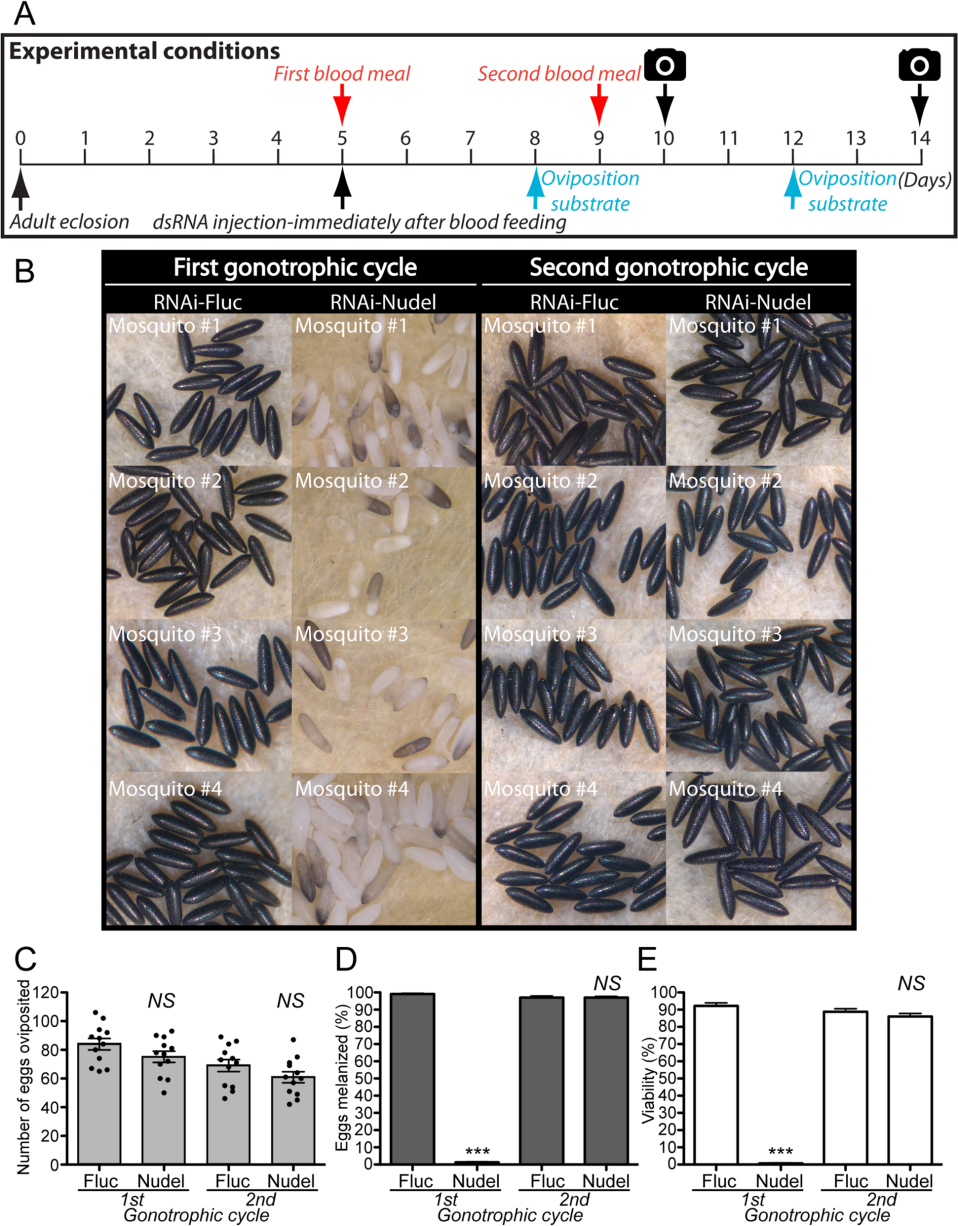
Eggs from the second gonotrophic cycle are not affected by dsRNA-Nudel when microinjected prior or during the blood feeding stage. **A** Schematic diagram of experimental time course for dsRNA microinjection, blood feeding, and oviposition in the first and second gonotrophic cycles. **B** Representative eggs are shown from mosquitoes microinjected with dsRNA-Fluc and dsRNA-Nudel. An effect of RNAi-Nudel and RNAi-Fluc control on fecundity (**C**), melanization (**D**), and viability (**E**) was examined during the first and second gonotrophic cycles. Each dot represents the number of eggs oviposited by an individual mosquito (**C**). Melanization was examined under a light microscope (**D**) and viability determined by hatching them 1 week after oviposition (**E**). Each bar corresponds to egg viability from 12 individual mosquitoes from three groups. The mean ± SEM are shown as horizontal lines. Statistical significance is represented by stars above each column (unpaired Student’s *t* test; *** *P* < 0.001, *NS* not significant). A detailed phenotypic analysis is shown in Additional file 7: Table S6

Next, we determined developmental- and tissue-specific expression patterns of Nasrat, Closca, Polehole, and Nudel during the first gonotrophic cycle in *A. aegypti* by qPCR. Similar adult female- and ovary-predominant expression patterns were observed for *Nasrat*, *Closca*, and *Polehole*. We observed 50 to 200-fold more transcripts in ovaries at 24 h PBM from these three genes compared to the sugar-fed fat body samples. The levels of mRNA encoding for these three eggshell structural proteins in the female reproductive organs increased only slightly in response to blood feeding (Fig. 3A–C). On the other hand, we found a high level of *Nudel* serine protease mRNA isolated from the whole body of 3-day-old adult male mosquitoes (Fig. 3D), while 200-fold upregulation of *Nudel* mRNA expression was detected in ovaries 36 h PBM in response to blood feeding. The expression dropped precipitously at the end of the primary follicle maturation phase (48 h PBM, Fig. 3D). We hypothesized that performing microinjection of dsRNA against these four proteins at specific time points may be important to the knockdown of corresponding mRNAs and may also play a role in inducing detrimental phenotypes of the eggs. Our results show that dsRNA microinjection against Nasrat, Closca, and Polehole immediately after blood feeding, rather than 3 days prior to blood feeding, did not result in defective egg phenotypes (Fig. 4A, B and Additional file 6: Table S5). This suggests that translation of these proteins occurred prior to blood feeding, ensuring their activity during eggshell formation and melanization. However for Nudel, even though egg production was not affected when dsRNA was microinjected immediately after blood feeding (Fig. 4C), egg melanization and viability were strongly impacted (Fig. 4D, E). This is likely due to the fact that Nudel mRNA transcription is tightly regulated in response to blood feeding, and suggests that Nudel may function in the late stage of eggshell formation. Thus, we tested whether an effect of RNAi knockdown of Nudel remains during the development of secondary follicles. Unlike the first gonotrophic cycle, defective phenotypes were no longer observed in eggs deposited by RNAi-Nudel females in the second gonotrophic cycle, and aspects of egg melanization and viability were not significantly affected (Fig. 5 and Additional file 7: Table S6). Similarly, abnormal egg phenotypes were not observed in mosquitoes with RNAi against Nasrat, Closca, and Polehole in the second gonotrophic cycle when mosquitoes were microinjected with dsRNA 1 day after adult eclosion. This is in contrast to our previous work showing that RNAi-mediated EOF1 depletion resulted in the formation of a defective eggshell throughout the first three gonotrophic cycles after a single dsRNA injection [34]. Taken together, the Nudel serine protease may interact with Nasrat, Closca, and Polehole structural proteins in the eggshell during the late follicle development stage. The consequence of this interaction may play key roles in the formation and melanization of *A. aegypti* mosquito eggshells.

### In vitro mosquito follicle melanization and permeability assays

**Fig. 6:**
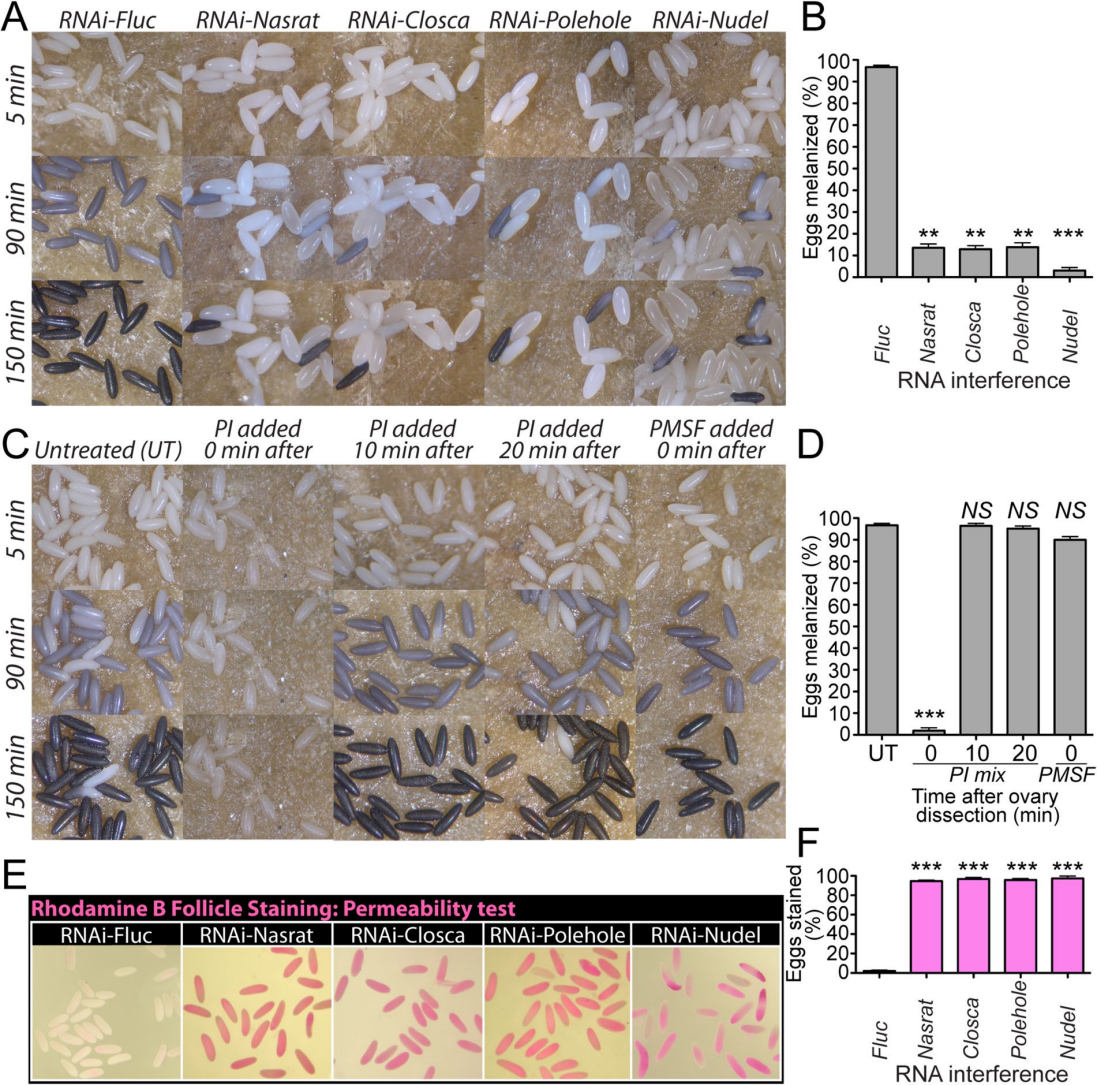
Nasrat, Closca, Polehole, and Nudel are essential for eggshell melanization and oocyte membrane permeability. **A** An in vitro follicle melanization assay was conducted using follicles isolated from gravid RNAi mosquitoes at 96 h PBM. Timing of dsRNA microinjection and blood feeding schedule was identical to those shown in Fig. 1. Follicles were photographed 5, 90 and 150 min after dissection. **B** Each bar corresponds to mean egg melanization (%) from five individual mosquitoes. The mean ± SEM are shown as horizontal lines. Statistical significance is represented by stars above each column (unpaired Student’s *t* test; *** *P* < 0.001, ** *P* < 0.01). **C** An in vitro follicle melanization assay was performed using a protease inhibitor cocktail (PI) to determine the role of proteases on eggshell melanization. The follicles were photographed 5, 90, and 150 min after follicle dissection. Follicles were incubated with PI (1X) at 0, 10, or 20 min after follicle dissection. Follicles were also incubated with PMSF (10 mM) immediately after dissection. **D** Each bar corresponds to mean egg melanization (%) from five individual mosquitoes. The mean ± SEM are shown as horizontal lines. Statistical significance is represented by stars above each column (unpaired Student’s *t* test; *** *P* < 0.001, *NS* not significant). **E** Follicle permeability assays were conducted using a Rhodamine B marker, with cellular uptake of Rhodamine B observed in primary follicles possibly due to a defective eggshell and oocyte plasma membrane. A detailed phenotypic analyses are shown in Additional file 8: Table S7 and Additional file 9: Table S8

Since RNAi-Nudel *A. aegypti* female mosquitoes laid melanization defective eggs, which led to embryonic death (Fig. 2), we hypothesized that the activation of the Nudel serine protease in wild type mosquitoes may be tightly regulated temporally and spatially in the perivitelline space. Additionally, we hypothesized that Nudel may be inactive prior to oviposition and that it is immediately activated upon oviposition, allowing Nudel to proteolytically regulate enzymes involved in melanization, sclerotization, and cross-linking of eggshells. To test our hypothesis, we developed an in vitro melanization assay for mosquito follicles to determine the time required for melanization of primary follicles isolated from RNAi mosquitoes. Mature follicles isolated from the ovaries of RNAi-Fluc controls 96 h PBM initiated melanization approximately 90 min after ovary dissection and completed the process approximately 150 min after ovary dissection (Fig. 6A). In contrast, most follicles isolated from mosquitoes treated with dsRNA against Nasrat, Closca, Polehole, and Nudel did not undergo melanization within the experimental period (Fig. 6A, B, and Additional file 8: Table S7). In fact, even 24 h after the experiments were initiated, melanization had not started.

To test whether active protease(s) are necessary during eggshell melanization, dissected mature primary follicles were soaked in vitro with a protease inhibitor (PI) cocktail (cOmplete™ Mini Protease Inhibitor Cocktail, Roche). Control follicles were soaked in water without PI. We observed that PI completely inhibited melanization of wild type mature follicles when treated during dissection (Fig. 6C, D). However, this effect was only observed if follicles were treated immediately after dissection. Follicles treated with PI at 10 or 20 min after dissection underwent normal eggshell melanization, similar to control follicles soaked in water only. These studies suggest that some eggshell proteins must be proteolytically processed immediately after exposure to water to successfully complete eggshell melanization. Next, we treated wild type primary follicles with phenylmethylsulfonyl fluoride (PMSF), an inhibitor of certain serine proteases. Interestingly, melanization was not blocked when follicles were treated at dissection with PMSF (Fig. 6C), suggesting that PMSF-sensitive serine proteases were not responsible for eggshell melanization, sclerotization, and cross-linking in *A. aegypti*.

Finally, we determined whether water permeability of the ovarian primary follicles is affected in dsRNA-treated mosquitoes with defective eggshells by staining the dissected follicles with rhodamine B. Rhodamine B permeability assays have an advantage that they can quickly assess whether follicles within the ovaries contain defective eggshells prior to oviposition. The representative images of follicles from RNAi assays are shown in Fig. 6E, F. Follicles from control RNAi-Fluc mosquitoes were only slightly stained by rhodamine B, while we observed increased permeability and uptake of rhodamine B through an oocyte plasma membrane in mosquitoes with RNAi knockdown of Closca, Polehole, and Nudel (Fig. 6E, Additional file 9: Table S8), indicating that these follicle could not properly regulate water permeability. Taken together, these data indicate that these four eggshell proteins play a significant role in eggshell formation and thus egg viability.

### Phenotypic characterization of eggs deposited by RNAi-DCEs and -CATL3 mosquitoes

**Fig. 7:**
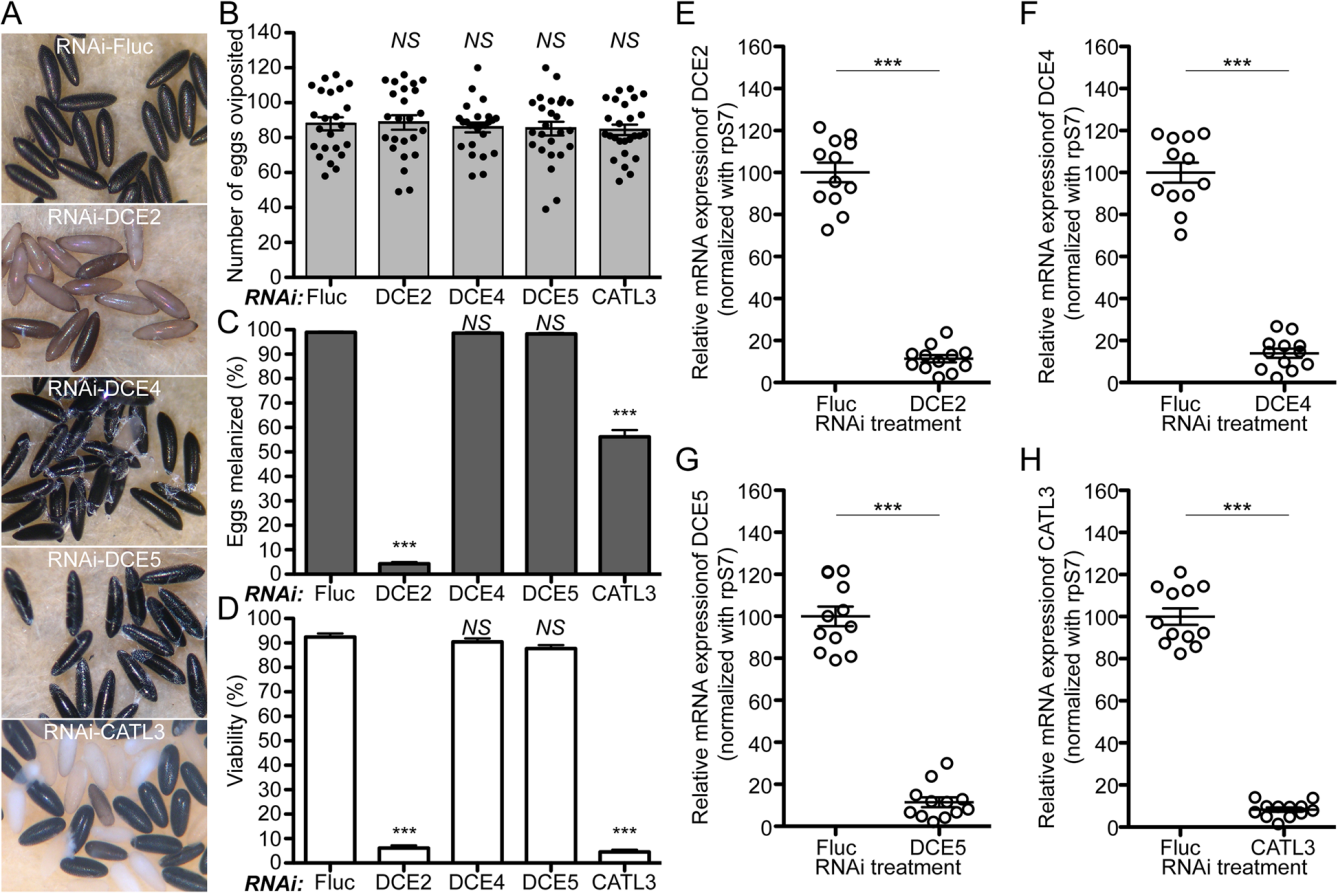
Reproductive phenotypes following RNAi knockdown of DCE2, DCE4, DCE5, and CATL3 in *Aedes aegypti* mosquitoes. **A** Representative eggs are shown from mosquitoes microinjected with dsRNA against Fluc control, DCE2, DCE4, DCE5, and CATL3. **B** An effect of RNAi on fecundity was studied during the first gonotrophic cycle. Each dot represents the number of eggs oviposited by an individual mosquito. **C** Melanization of these eggs was examined under a light microscope and a melanization percentage was determined. **D** An effect of RNAi on viability of eggs was examined by hatching eggs 1 week after oviposition. Each bar corresponds to egg viability from 23 to 26 individual mosquitoes from three independent cohorts. The mean ± SEM are shown as horizontal lines. Statistical significance is represented by stars above each column (unpaired Student’s *t* test; *** *P* < 0.001, *NS* not significant). A detailed phenotypic analysis is shown in Additional file 10: Table S9. RNAi-mediated knockdown efficiency was validated by quantitative real-time PCR (qPCR). Relative abundance of mRNA levels for DCE2 (**E**), DCE4 (**F**), DCE5 (**G**), and CATL3 (**H**) was analyzed in dissected mosquito ovaries at 36 h PBM. Mosquitoes were microinjected with each dsRNA at 4 days prior to blood feeding, as shown in Fig. 1. dsRNA-Fluc-injected mosquitoes were used as controls. A single mosquito analysis was performed to isolate total RNA, synthesize cDNA, and monitor silencing efficiency by qPCR. mRNA levels were normalized according to transcript levels of ribosomal S7 protein. Data are presented as MEAN ± SEM of 12 individual mosquitoes. *** *P* < 0.001 compared to RNAi-Fluc

In addition to four proteins studied above, RNAi screening identified four mosquito eggshell proteins, DCE2, DCE4, DCE5, and CATL3, necessary to form intact eggshells (Fig. 1). We performed RNAi studies to further characterize reproductive phenotypes, including fecundity, egg melanization, and viability in the first gonotrophic cycle. Representative egg phenotypes from RNAi treated mosquitoes are shown in Fig. 7A. RNAi-mediated loss of either DCE2, DCE4, DCE5, or CATL3 did not significantly affect egg production, compared to the RNAi-Fluc control (Fig. 7B, Additional file 10: Table S9). RNAi silencing of DCE2 and CATL3 had differential effects on eggshell melanization. Approximately 96% of total eggs from RNAi-DCE2 females were only partially melanized, while 43% of eggs from RNAi-CATL3 females were non-melanized (Fig. 7C, Additional file 10: Table S9). In contrast, nearly all eggs from RNAi-DCE4 and -DCE5 mosquitoes were melanized normally, but had readily observable defective eggshell surface phenotypes. Approximately 95% of eggs from RNAi-DCE2 and -CATL3 females were non-viable, while nearly 90% of laid by RNAi-DCE4 and -DCE5 mosquitoes produced viable first instar larvae (Fig. 7D, Additional file 10: Table S9). qPCR using gene-specific primers (Additional file 5: Table S4) validated that RNAi against *DCE2*, *DCE4*, *DCE5*, and *CATL3* had significant effects on the reduced level of the corresponding endogenous mRNA in the ovaries (Fig. 7E–H).

**Fig. 8:**
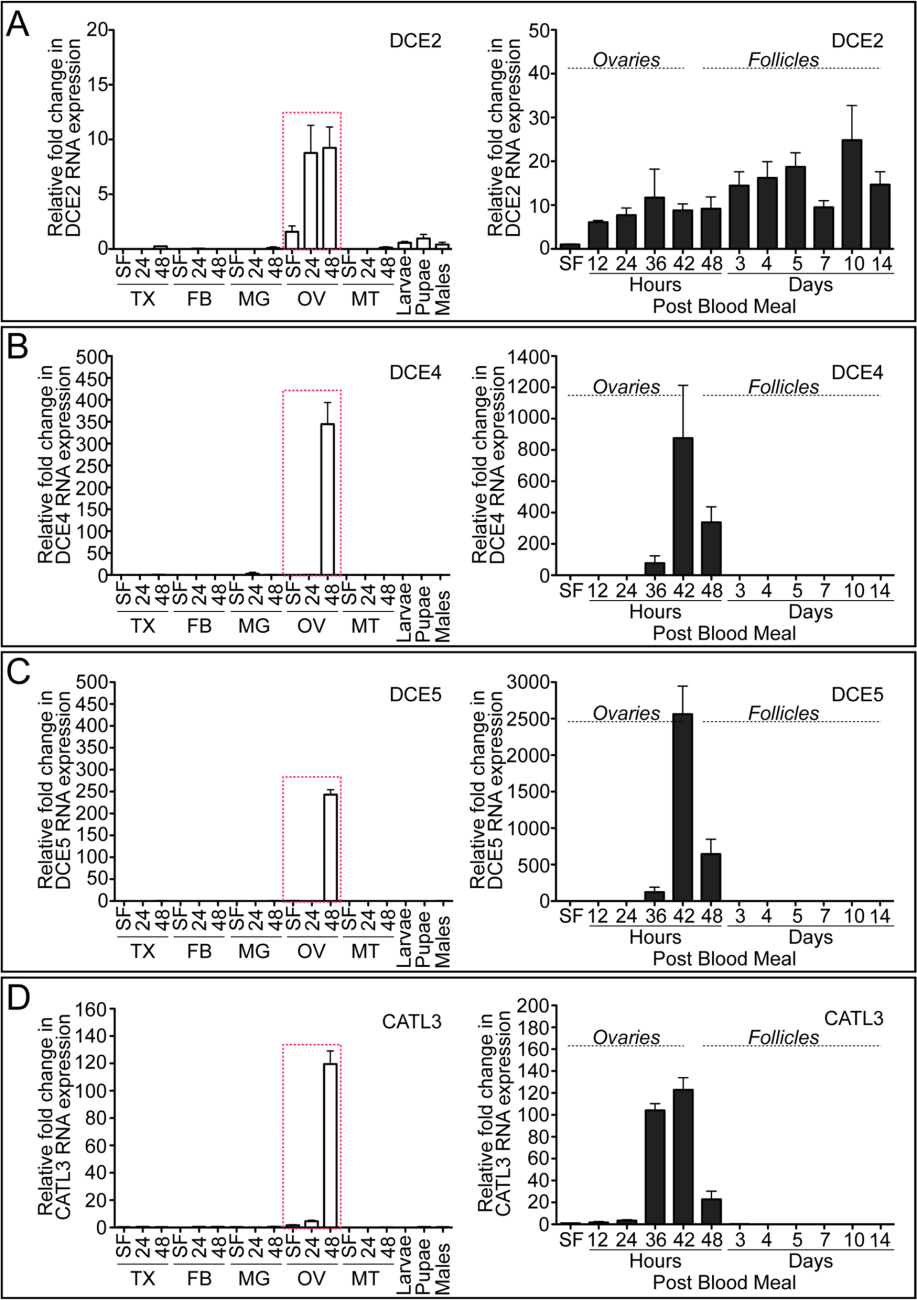
Transcript expression of DCE2, DCE4, DCE5, and CATL3 during the first gonotrophic cycle in *Aedes aegypti* mosquitoes. Tissue-specific and developmental expression patterns of DCE2 (**A**), DCE4 (**B**), DCE5 (**C**), and CATL3 (**D**) during the first gonotrophic cycle are shown in the left graphs. Gene expression was analyzed by qPCR using cDNAs prepared from various tissues. Tissues included are thorax (TX), fat body (FB), midgut (MG), ovary (OV), and Malpighian tubules (MT) in sugar-fed only (SF), 24, and 48 h PBM, as well as larvae (Lv), pupae (Pp), and adult males (M). The expression in fat body at SF was set to 1.0. DCE2, DCE4, DCE5, and CATL3 were predominantly expressed in ovaries (highlighted with a red dotted rectangle). In addition, a detailed transcript expression study in mosquito ovaries during the first gonotrophic cycle was also performed. In the right-hand plots, samples from SF to 36 h PBM include entire ovaries, whereas those from 48 h to 14 days PBM include only primary follicles isolated from ovaries. Expression in ovaries at SF was set to 1.0. The expression levels for DCE2, DCE4, DCE5, and CATL3 were normalized to S7 ribosomal protein transcript levels in the same cDNA samples. Data were collected from three different mosquito cohorts. The mean ± SEM are shown as horizontal lines

Expression of *DCE2*, *DCE4*, *DCE5*, and *CATL3* transcript expression during the first gonotrophic cycle exhibited clear adult female- and ovary-specific expression patterns (Fig. 8). A fivefold increase in *DCE2* ovarian mRNA was observed in response to blood feeding, with maximal expression occurring 36 h PBM (Fig. 8A). *DCE4* and *DCE5* mRNA expression patterns were similar to each other and highly induced in ovaries after blood feeding, peaking at around 42 h PBM (Fig. 8B, C). *CATL3* transcripts were highly upregulated in ovaries between 36 and 42 h PBM (Fig. 8D). Transcripts encoding *DCE4*, *DCE5*, and *CATL3* were no longer observed by 72 h PBM, suggesting that their expressions may be exclusively present in the follicular epithelial cells that undergo shedding and apoptosis when follicles are ready for oviposition.

### Ultrastructural analysis of defective eggshell phenotypes

**Fig. 9:**
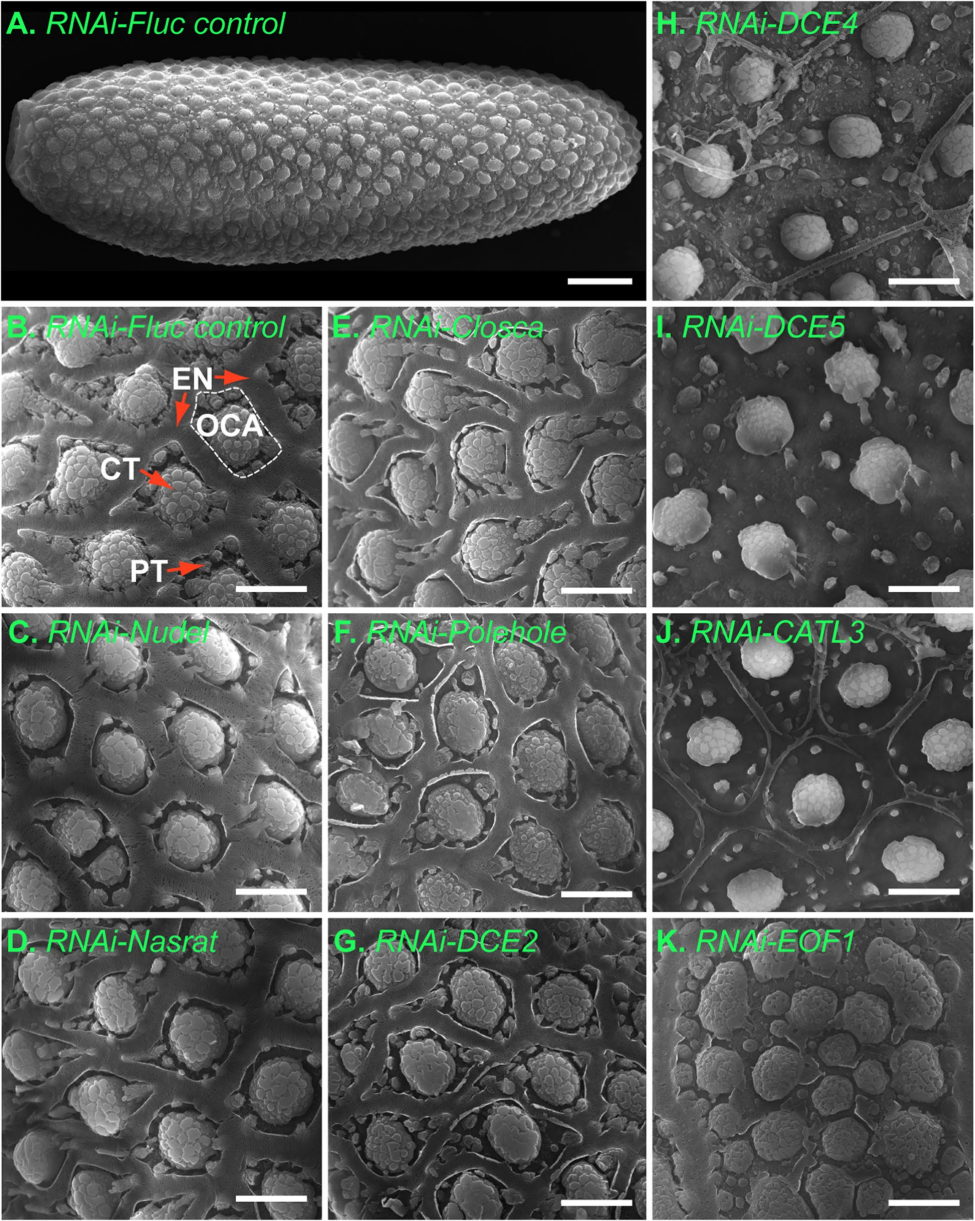
Scanning electron micrograph images of eggshell ultrastructural features from RNAi *Aedes aegypti* mosquitoes. **A** Dorsal view of an entire primary follicle from an RNAi-Fluc mosquito. The image was taken under × 600 magnification (scale bars = 50 μm). **B** Representative dorsal images are shown from RNAi-Fluc control (**B**), -Nudel (**C**), -Nasrat (**D**), -Closca (**E**), -Polehole (**F**), DCE2 (**G**), -DEC4 (**H**), -DCE5 (**I**), -CATL3 (**J**), and -EOF1 (**K**) mosquitoes. The photos were taken under × 6000 magnification (scale bars = 10 μm). CT, central tubercle; PT, peripheral tubercle; EN, exochorionic network; OCA, outer chorionic area. Mosquitoes were injected with dsRNA as in Fig. 1. Mature primary follicles were dissected in 1 × PBS at 96 h PBM, immediately fixed with 2.5% electron microscopy grade glutaraldehyde at 4 °C, post-fixed with 1% osmium tetroxide at room temperature, dehydrated with 100% ethanol, dried with hexamethyldisilazane, and metallized with gold. All dorsal images of eggs were taken using a FEI Inspect-S electron scanning microscope at the W.M. Keck Center for Nano-Scale Imaging, University of Arizona (RRID:SCR_022884)

We characterized the effect of RNAi on topological surface features of the mosquito eggshell in detail using scanning electron microscopy (SEM). SEM images of an entire primary follicle and its detailed surface features from RNAi-Fluc control samples are shown, respectively, for comparison (Fig. 9A, B). Ultrastructural analysis of the eggshell exochorion from *A. aegypti* has been previously conducted [37–40]. Key features are shown in Fig. 9B, including the outer chorionic area (OCA) surrounded by the porous exochorionic network (EN), protruding central tubercles (CT), and several minute peripheral tubercles (PTs). Using these structures as a guide, we examined eggs that exhibited defective phenotypes during RNAi screening of eggshell proteins (Fig. 1), as well as Nudel, Polehole, and EOF1. Nudel serine protease and Nasrat, Closca, and Polehole structural proteins intimately interact with one another and RNAi knockdown of these proteins led to reduced melanization phenotypes (Fig. 2). Surprisingly, SEM analysis showed that the exochorionic surface topology of eggs from RNAi-Nudel, -Nasrat, -Closca, and -Polehole females did not differ dramatically from RNAi-Fluc controls (Fig. 9C–F), even though eggs from these cohorts had defective eggshell melanization phenotypes. Furthermore, while RNAi-DCE2 eggs were also incompletely melanized (Fig. 1), SEM analysis found no abnormal surface phenotypes (Fig. 9G). These SEM studies suggest that these five eggshell proteins may impact the melanization of an endochorion layer beneath the exochorion. On the other hand, RNAi against DCE4 and DCE5 resulted in phenotypes in which the outermost exochorion appears to be partially peeled or entirely absent (Fig. 1). SEM images confirmed that a portion of the EN was greatly reduced and in some cases peeled away from the exochorion, while PTs seem greatly reduced in both number and size for DCE4, DCE5 and CATL3 (Fig. 9H–J). Thus, DCE4 and DCE5 enzymes may be responsible for synthesizing or cross-linking the EN to the exochorion surface. Eggs from CATL3-deficient mosquitoes are more oval-shaped than control mosquitoes (Fig. 1 and Fig. 7), and RNAi-CATL3 led to deformed EN structures under SEM (Fig. 9J). However, these ultrastructural features (Fig. 9B–J) are dissimilar to eggshell surface characteristics resulting from RNAi-EOF1 (Fig. 9K, [34]), where enlarged OCAs contain multiple CTs. Since a loss of EOF1 function by RNAi led to altered exochorion ultrastructural features and defects in endochorion melanization, the mosquito-specific intracellular EOF1 protein may regulate multiple target proteins that are independently responsible for surface patterning of exochorion layer and melanization of endochorion layer in *A. aegypti* mosquitoes*.*

### Proteomic analysis on mosquito eggshells

**Fig. 10:**
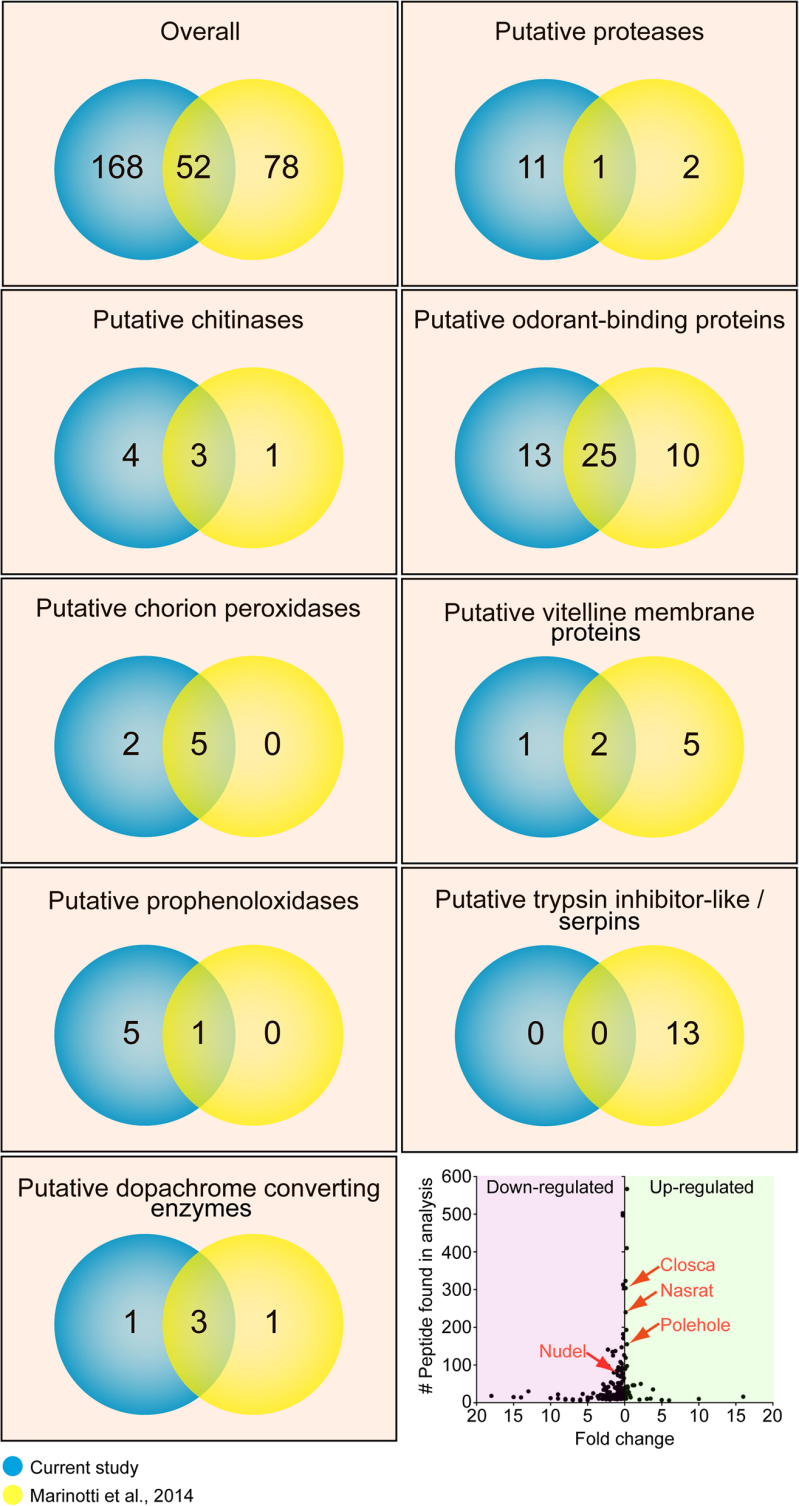
Venn diagram illustrating the identification of proteins in different eggshell proteomic data sets. Venn diagram comparing the number of identified eggshell proteins between the current study and previously published data by Marinotti et al. [6]. About 40% of the eggshell proteins from the previous study were found in the current study, while 168 additional proteins were detected from our eggshell proteomic analysis. Detailed identified proteins are listed in Additional file 11: Table S10. Eight putative protein functions were evaluated by comparing identified eggshell proteins from this study with previously published proteomics data by Marinotti et al. [6]. Proteins with more than or equal to six peptide hits from four independent proteomic data sets were included. Two independent biological replicates from both RNAi-Fluc and RNAi-EOF1 were used in the proteomic analysis. Eggshell peptide abundance fold changes in response to RNAi-EOF1 (AAEL012336) are shown in comparison with RNAi-Fluc control

Extracellular eggshell structural proteins and enzymes have been identified in previous mosquito eggshell proteomic analyses [6, 28]. Specifically, enzymes including chitinases, chorion peroxidases, prophenoloxidases, dopachrome-converting enzymes, laccase-like multicopper oxidase, proteases, and others have been found and recognized for their importance in eggshell formation. To determine whether EOF1 deficiency in mosquitoes leads to changes in the expression of eggshell target proteins, we performed proteomic analysis of eggshells derived from RNAi-Fluc control and RNAi-EOF1 mosquitoes. Our current study employed a novel approach that used Dounce homogenizers to enrich extracellular eggshells from ovarian follicle cells. Eggshell proteins were subsequently extracted with guanidine hydrochloride. Our current mass spectrometry analysis detected a total of 220 eggshell proteins (Fig. 10, Additional file 11: Table S10), which included 168 novel proteins, confirmed the presence of 52 proteins identified in the previous proteomics study and failed to identify 77 proteins previously identified from the *A. aegypti* eggshell [6]. This suggests that different eggshell isolation and protein extraction methods may be ideally suited for enriching specific eggshell proteins.

Numerous enzymes are involved in insect eggshell formation [27]. Eight putative enzyme and protein functions were further evaluated by comparing previously published proteomics data [6] with eggshell proteins identified in this study (Fig. 10). Extracellular chorion peroxidases have been implicated in the formation of rigid, insoluble eggs by catalyzing an eggshell protein cross-linking reaction [21, 41, 42]. In our analysis, we found four additional proteins possessing chitinase domains and discovered two chorion peroxidases in addition to the five detected previously [6]. Together, these data suggests that these enzymes may facilitate eggshell hardening to protect fertilized oocytes undergoing embryogenesis. Similarly, we identified five new putative prophenoloxidases, which may play key roles in the process of eggshell melanization in mosquitoes [41]. Insect DCEs have been studied in *A. aegypti* [22, 23], *D. melanogaster* [43], *Tribolium castaneum* [44], and *Musca domestica* [45], since they are responsible for catalyzing melanization and sclerotization reactions. We now know that at least five extracellular DCE assist in the formation of intact eggshells in *A. aegypti* and likely have specific functions (Fig. 10). For example, DCE2 is required for melanization (Fig. 7A), while DCE4 and DCE5 play a role in the formation of the fine EN structures that define OCA at the eggshell exochorion (Fig. 9H, I). We identified 11 new eggshell proteases including Nudel, but surprisingly we did not detect CATL3, which was found in a previous eggshell proteomics study [6] and is an essential cysteine protease for egg viability as demonstrated in this study (Fig. 7). Additional RNAi knockdown studies against newly identified proteases may reveal their essential functions in mosquito eggshells. The mass spectrometry data from previous studies and our current study combined have identified 48 putative odorant-binding proteins with unknown functions in the *A. aegypti* eggshell (Fig. 10, Additional file 11: Table S10). The previous study identified seven vitelline membrane proteins, while in the current proteomics study we detected two of those proteins along with one novel protein. Interestingly, our eggshell isolation strategy failed to detect any putative trypsin inhibitor-like proteins or serpins. Finally, we did not observe a dramatic effect of RNAi-EOF1 on peptide abundance for Nasrat, Closca, Polehole, Nudel, DCE2, DCE4, and DCE5, relative to RNAi-Fluc control (Fig. 10, Additional file 11: Table S10).

It is possible that EOF1 deficiency results in the mislocalization of these proteins without affecting expression levels, while still leading to the defective phenotypes. Alternatively, other newly identified uncharacterized eggshell proteins (Additional file 11: Table S10) may be essential for melanization and cross-linking processes and downstream targets of EOF1.

Pathogen transmission by mosquito vectors poses a significant problem on a global scale. However, current mosquito control strategies are largely ineffective due to increasing insecticide resistance and a lack of novel control strategies. One such novel approach to controlling mosquito populations is to disrupt specific molecular processes or antagonize novel metabolic targets required to produce viable eggs. Egg production is a carefully regulated series of events that offers numerous opportunities for control. During the late stages of egg development, eggshell formation represents a critical step that ensures the viability of the developing embryo.

## Discussion

Pathogen transmission by mosquito vectors poses a significant problem on a global scale. However, current mosquito control strategies are largely ineffective due to increasing insecticide resistance and a lack of novel control strategies. One such novel approach to controlling mosquito populations is to disrupt specific molecular processes or antagonize novel metabolic targets required to produce viable eggs. Egg production is a carefully regulated series of events that offers numerous opportunities for intervention. During the late stages of egg development, eggshell formation represents a critical step that ensures the viability of the developing embryo. The eggshell extracellular matrix located between the follicular epithelial cells and the oocyte is formed in the ovaries prior to oviposition, while eggshell melanization and cross-linking occur following oviposition to protect the recently laid embryo from harsh environmental conditions. A fully developed *A. aegypti* mosquito eggshell can protect the first instar larva from desiccation and physical damage for over a year prior to hatching once the arrested egg is submerged in water. In this study, we identified multiple eggshell proteins and enzymes that were found to be necessary for successful egg development through a comprehensive series of RNAi assays that identified and characterized key eggshell phenotypes in *A. aegypti*.

**Fig. 11:**
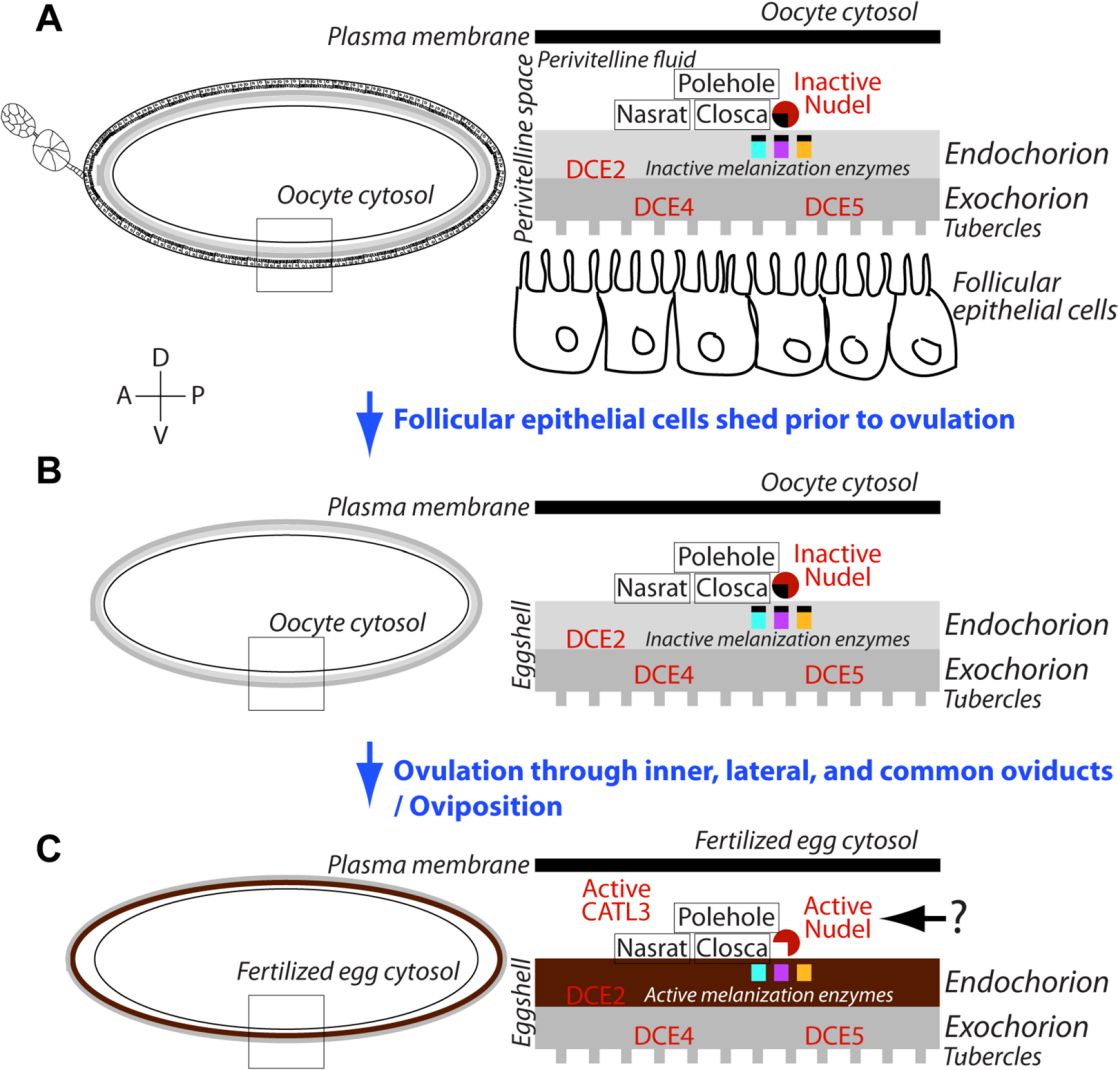
A proposed schematic model for the involvement of specific proteins during eggshell formation and melanization in *Aedes aegypti* mosquitoes. **A** A primary follicle in the ovariole reaches maturity and an extracellular eggshell matrix forms between the oocyte and follicular epithelial cells around 48 h PBM. Enzymes involved in eggshell melanization and cross-linking are present but inactive during this stage. **B** As primary follicles initiate migration into an inner oviduct, the surrounding follicular epithelium sheds with the secondary follicle and germarium in the ovariole. The enzymes involved in eggshell melanization and cross-linking are still in their inactive forms within the inner, lateral, and common oviducts. **C** Once the eggs are deposited onto an oviposition substrate, eggs briefly uptake surrounding water and activate the melanization and cross-linking enzymes through the activity of Nudel and/or CATL3 leading to tanning and completion of the eggshell

**Fig. 12:**
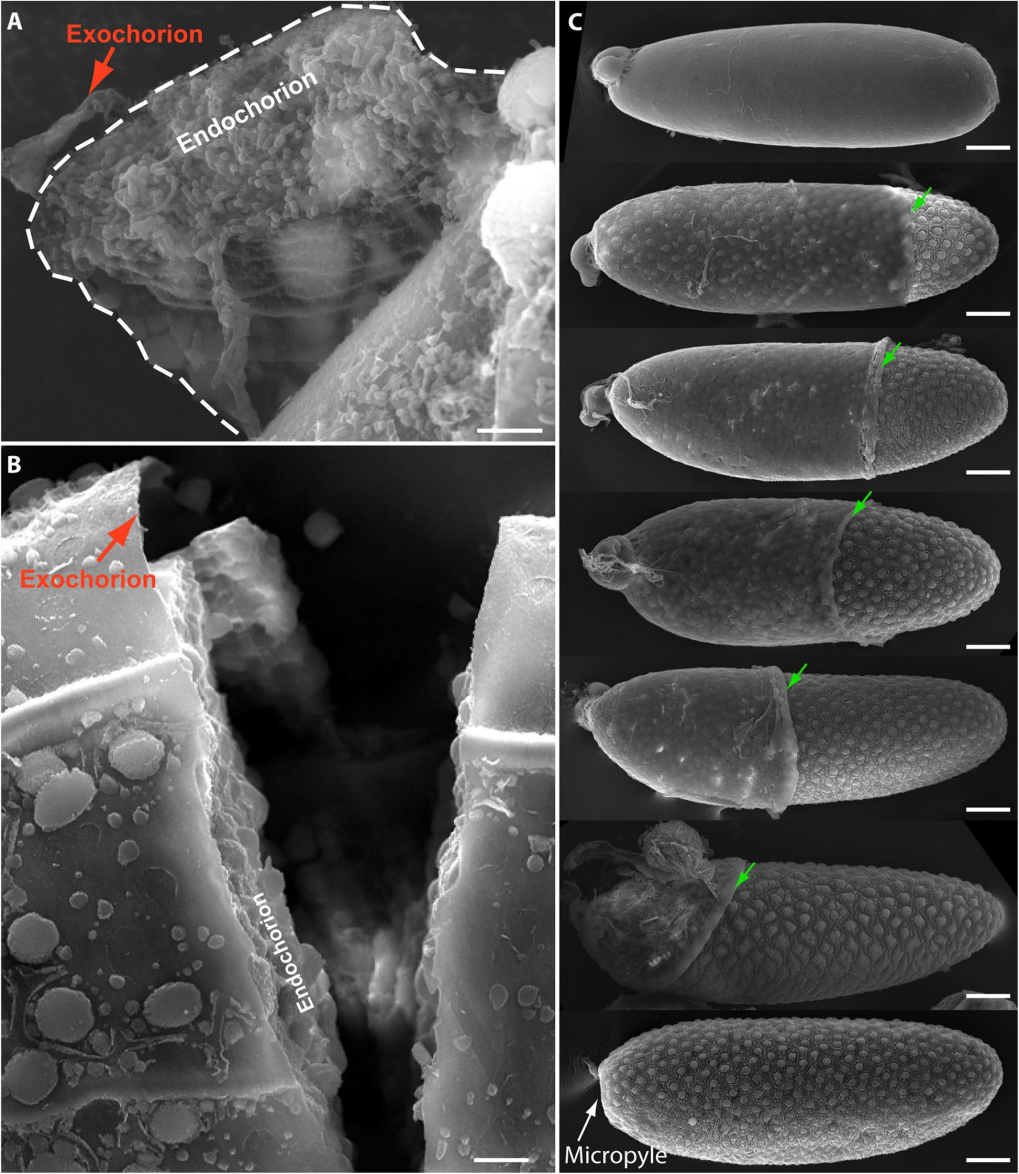
Scanning electron micrograph images of late follicle development in *Aedes aegypti*. **A** SEM image shows a peeled area of exochorion and endochorion, exposing the complex internal eggshell structures. **B** SEM image shows a vertical breakage of mosquito egg, exposing a very thin exochorion and the complex endochorion eggshell structures. **C** Scanning electron micrograph images of individual follicles during late-oogenesis, showing gradual shedding of the follicular epithelial cell layer from the posterior to anterior direction. Programmed shedding of follicular epithelial cells, secondary follicles, and germarium from the primary follicle in the ovariole occurs during late oocyte development prior to oviposition. Green arrows indicate the leading edge of the follicular epithelial cell layer. Follicles were derived from wild type mosquitoes and prepared as described in Fig. 9 and in the materials and methods. Images were obtained using an FEI Inspect-S electron scanning microscope at the W.M. Keck Center for Nano-Scale Imaging, University of Arizona (RRID:SCR_022884). Photos were taken under × 6000 magnification (**A**; scale bar 5 μm), under × 1200 magnification (**B**; scale bar 10 μm), and under × 500 magnification (**C**; scale bars 50 μm)

A proposed summary model of eggshell formation and melanization in *A. aegypti* mosquitoes is shown in Fig. 11. As the primary follicle reaches maturity following the uptake of sufficient vitellogenin yolk proteins around 48 h PBM, the secondary follicle attached to a germarium remains dormant in the resting stage. The extracellular eggshell matrix is sequentially built between the nearly mature oocyte and follicular epithelial cells that surround the oocyte and nurse cells, with the eggshell matrix being completely formed by 48 h PBM. SEM images reveal the complex structures of the exochorion (Fig. 9) and endochorion (Fig. 12A, B). Previously, we discovered that EOF1 was essential for eggshell formation and melanization in both *A. aegypti* and *A. albopictus* mosquitoes [34]. Since EOF1 was not predicted to contain a secretory signal sequence and was not identified in previous mosquito eggshell proteomic studies [6, 28], we hypothesized that EOF1 was an intracellular protein that regulated the secretion and activation of unknown eggshell proteins during ovarian follicle maturation. To test this hypothesis, we performed systematic RNAi screening of previously identified eggshell proteins [6] and identified several target eggshell proteins that may act downstream of EOF1. The eggshell proteins Nasrat, Closca, and Polehole are thought to form a heterotrimeric complex and localize at the perivitelline fluid side of the endochorion, as shown in *D. melanogaster* [35, 36]. The Nudel serine protease accumulates at high levels and is tethered to this complex during late follicle development. The disrupted melanization and greatly reduced larval viability observed following knockdown of Nudel, Nasrat, Closca, and Polehole indicate that complete eggshell melanization correlates with egg viability and that these four proteins likely function together to regulate molecular and biochemical pathways leading to eggshell melanization in the mosquito.

DCE proteins also play a critical role during this period of eggshell formation with DCE2 possibly being localized at the endochorion, while DCE4 and DCE5 were localized to the exochorion. Roles of DCE (yellow) proteins have also been studied from *A. albopictus* eggs [46, 47]. Additional enzymes responsible for melanization, sclerotization, and cross-linking are believed to be present as inactive forms in their respective locations within the eggshells; and these become active shortly after oviposition. Once the primary follicles are ready for ovulation and ovipositon, the layer of follicular epithelial cells surrounding the eggshell, along with the secondary follicle and germarium, undergo a shedding process. The follicular epithelial cells are peeled off the mature primary follicle beginning from the posterior end of the egg and progressing to the anterior end (Fig. 12C). The secondary follicle takes a position at the space for the existed primary follicle and now becomes competent to uptake vitellogenin yolk proteins after blood feeding, while the germarium produces tertiary follicle. Following shedding of the follicular epithelial cells, the mature primary follicles move through inner, lateral, and common oviducts before the fertilized eggs are eventually deposited onto a wet oviposition substrate. Surrounding water is instantaneously and briefly absorbed by the eggs before the eggs become impermeable to water. Both Nudel and CATL3 in eggshells are activated through unknown mechanisms; however, once they are activated, they proteolytically activate downstream enzymes involved in the eggshell melanization and cross-linking processes.

In this study, we identified eight essential eggshell proteins, Nasrat, Closca, Polehole, Nudel, DCE2, DEC4, DCE5, and CATL3, through RNA interference screening and determined their egg phenotypes including fecundity, melanization, and viability. All eight characterized eggshell proteins were required for intact eggshell formation and were predominantly expressed in the ovaries of adult females. Because all these proteins may predominantly be expressed and secreted by the follicular epithelial cells, which surround a mature oocyte and undergo a shedding process prior to the departure of the mature primary follicles from an ovariole into an inner oviduct, the secondary follicles and their associated immature state of follicular epithelial cells may not be adversely affected when dsRNA microinjection occurs during the first gonotrophic cycle. Furthermore, Nasrat, Closca, Polehole, Nudel, and DCE2 were found to be responsible for maintaining the integrity of eggshell, and in turn embryo viability. Interestingly, the melanization process did not affect exochorionic ultrastructures. Conversely, the functions of DEC4, DCE5, and CATL3 are coupled to the dynamic exochorionic surface topology of mosquito eggs. Surprisingly, the changes to the eggshell surface topologies in response to RNAi knockdown of these proteins did not phenocopy the effect of RNAi-EOF1. Thus, it remains unclear how EOF1 influences eggshell formation and melanization. In this study, our eggshell proteomics identified an additional eggshell protein from *A. aegypti* mosquitoes. We used guanidine hydrochloride, which is a strong chaotropic reagent, to remove proteins that were tightly cross-linked to eggshell components including chitin, which is polymer of N-acetyl-glucosamine. The proteins were also differentially extracted from the protein gels, making it possible to increase the number of proteins recovered. Proteomic analysis of eggshell proteins from RNAi-EOF1 led to the identification of additional proteins that could be potential targets of EOF1 during eggshell formation and additional studies are needed to characterize these candidates. Understanding reproductive processes of critical insect vectors of human disease could lead to the development of selective and safe small molecular inhibitors that may act to reduce the rate of disease transmission and these new data lay the groundwork for identifying novel control targets in *A. aegypti*.

## Conclusions

In conclusion, our comprehensive RNAi screening studies of putative eggshell proteins resulted in the identification of eight additional proteins involved in eggshell formation in *A. aegypti* mosquitoes. These ovary-specific proteins are essential for proper eggshell formation and melanization and egg viability. Our findings may provide new insights into new mosquito control strategies.

## Methods

### Mosquitoes

*A. aegypti* (Rockefeller strain) were reared at 28 °C, 80% relative humidity, and a 16-h light and 8-h dark cycle. Larvae in water were fed on a diet containing dry dog food, fish flakes, and liver powder (10:10:1 weight ratio). Adult female mosquitoes were allowed to feed on blood supplemented with fresh adenosine triphosphate (5.0 mM final concentration) through stretched parafilm on an artificial glass feeder. Only fully engorged females identified under a light microscope were used. Human blood was donated by the American Red Cross (Tucson, AZ). Adult mosquitoes were also provided 10% sucrose ad libitum.

### RNAi screening of eggshell genes in *A. aegypti*

Target eggshell proteins were chosen from a previously published proteomic analysis [6]. RNAi was performed to silence mRNA encoding mosquito eggshell genes (Additional file 1: Table S1). Each gene-specific forward and reverse oligonucleotide primer was designed using a NetPrimer web-based primer analysis tool. T7 RNA polymerase promoter sequence, TAATACGACTCACTATAGGGAGA, was placed to the 5′ end of each primer (Additional file 2: Table S2). All primers were obtained from Eurofins Genomics (Louisville, KY). A total RNA was isolated from mosquito whole body using Trizol Reagent (Invitrogen, Carlsbad, CA), and subsequently complementary DNA (cDNA) was synthesized using M-MLV reverse transcriptase (Promega, Madison, WI). PCR was performed using Taq 2X Master Mix (NEB, Ipswich, MA), and the amplified PCR products were ligated into the pGEM-T easy vector (Promega) for DNA sequence verification. dsRNA was synthesized by in vitro transcription using the PCR products as templates, NTP nucleotides, and T7 RNA polymerase from HiScribe™ T7 Quick High Yield RNA Synthesis Kit (NEB). dsRNA was resuspended in HPLC-grade water at 7.5 μg/μL. Cold-anesthetized female mosquitoes were microinjected with 2.0 μg (276 nL) gene-specific dsRNA using a Nanoject II microinjector (Drummond Scientific Company, Broomall, PA). Mosquitoes were maintained on 10% sucrose following injection. RNAi mosquitoes were allowed to mate and fed a blood meal 4 days after dsRNA microinjection. Fully engorged females were individually placed into oviposition containers. Eggs on oviposition paper were photographed 2 days post oviposition under a light microscope (Nikon, SMZ-10A). Phenotypes including the number and percent melanization of eggs were recorded. To determine egg viability, eggs on oviposition papers remained wet for 3 days to allow for the completion of embryonic development before drying the eggs at 28 °C. Seven days after injection, the eggs were submerged in water, placed under a vacuum using a Speed Vac for 10 min, and allowed to hatch over 2 days.

### Assessing eggshell gene expression using qPCR

We determined the expression patterns of four eggshell proteins, Nasrat, Closca, Polehole, and Nudel. Tissue samples were obtained from larvae, pupae, and male adults, as well as in five female tissues (thorax, fat body, midgut, ovaries, and Malpighian tubules) from sugar-fed (3 days post-eclosion) and blood-fed (24 and 48 h PBM) mosquitoes. Tissues were dissected in 1X PBS under a light microscope and transferred into tubes containing Trizol. Fourth instar larvae and pupae of mixed age and sexes were thoroughly washed with H_2_O before placing them in TRIzol reagent (Invitrogen). Total RNA was extracted according to the manufacturer’s instructions. First-strand cDNA was synthesized from DNaseI-treated total RNA using an oligo-(dT)_20_ primer and M-MLV reverse transcriptase (Promega). qPCR was carried out with the corresponding cDNA, gene-specific primers (Additional file 5: Table S4), PerfeCTa SYBR Green FastMix, and ROX (Quanta BioSciences, Gaithersburg, MD) on a 7300 Real-Time PCR System (Applied Biosystems, Waltham, MA). Relative expression for Nasrat, Closca, Polehole, and Nudel was calculated as 2^−ΔΔCt^ and normalized to ribosomal protein S7 transcript levels in the same cDNA samples. Data were collected from 10 mosquitoes (three different biological cohorts).

### Measuring knockdown efficiency of eggshell genes at the transcription level

The level of RNAi-mediated transient knockdown was verified by qPCR using gene-specific primers (Additional file 5: Table S4). cDNA was synthesized from DNaseI-treated total RNA derived from ovaries of individual dsRNA-injected mosquitoes at 36 h PBM followed by qPCR as described above. Relative expression for Nasrat, Closca, Polehole, Nudel, DCE2, DCE4, DCE5, and CATL3 was calculated as 2^−ΔΔCt^ and normalized to ribosomal protein S7 transcript levels in the same cDNA samples. Knockdown efficiency was compared using Fluc dsRNA-injected mosquitoes as a control. Data were collected from 12 individual mosquitoes.

### In vitro follicle melanization assay

An in vitro follicle melanization assay was performed using mature primary follicles isolated from the ovaries of gravid RNAi mosquitoes (RNAi-Fluc, -Nasrat, -Closca, -Polehole, and -Nudel) at 96 h PBM. The timing of dsRNA microinjection and blood feeding schedule are detailed in Fig. 1. Follicles were placed on oviposition paper wetted with distilled water and photographed at 5, 90, and 150 min after dissection. Follicles from five individual mosquitoes were used. Dissected primary follicles were also soaked in vitro with PI (1 × concentration in water; cOmplete™ Mini Protease Inhibitor Cocktail, Sigma). Ovaries from female mosquitoes at 96 h PBM were dissected and placed in water containing PI at different time points after ovary dissection. Individual follicles were then separated, transferred onto oviposition paper, continuously soaked with water containing the PI, and monitored for eggshell melanization. Control follicles were soaked in water without PI. Follicles were also treated with PMSF at a 10 mM final concentration.

### Rhodamine B mosquito follicle permeability assay

The advantage to using a Rhodamine B assay is that it can quickly assess whether follicles within the ovaries may contain a defective eggshell prior to oviposition. Ovaries of RNAi mosquitoes were dissected at 96 h PBM, and individual primary follicles were separated from the ovaries and transferred to glass scintillation vials containing Rhodamine B (Sigma) at a 1.0 mM final concentration in water. The follicles were stained with Rhodamine B for 10 min on a rocking shaker and thoroughly rinsed with water 5 times every 10 min. The follicles were photographed under a light microscope with a Coolpix 4300 camera (Nikon).

### Ultrastructural study of eggshell by SEM

Ovaries were dissected from mosquitoes that had undergone microinjection with different dsRNAs at 96 h PBM. Mature primary follicles were separated from the ovaries in 1 × PBS under a light microscope. The follicles were washed several times with 1 × PBS and fixed with 2.5% EM-grade glutaraldehyde (EMS, Electron Microscopy Sciences, Hatfield, PA) in 0.1 M PIPES (pH 7.2) overnight at 4 °C prior to being washed thoroughly with 0.1 M PIPES. The follicles were then post-fixed in 1% osmium tetroxide (EMS) in 0.1 M PIPES for 1 h in the dark, and subsequently washed thoroughly in deionized water. Next, the follicles were dehydrated with ethanol (ETOH) in water through a graded series for 10 min each in 10, 30, 50, 70, and 90% ETOH and three times for 30 min each in 100% ETOH at room temperature. The dehydrated follicles were then stored at 4 °C for 4 days. The samples were dried with hexamethyldisilazane (HMDS, EMS) in ETOH through a graded series for 20 min each in 25, 50, 75, and 100% HMDS at room temperature. Finally, follicle samples were air-dried under a fume hood overnight at room temperature prior to SEM analysis. The dried samples were metallized with gold using Hummer 6.2 Sputter System (Anatech USA, Union City, CA). An Inspect-S scanning electron microscope (FEI, Hillsboro, OR) was used to compare the ultrastructural surface features of the follicles isolated from various RNAi mosquitoes at the W.M. Keck Center for Nano-Scale Imaging, University of Arizona (RRID:SCR_022884).

### Mosquito eggshell protein extract preparation

Mosquitoes were microinjected with dsRNA against Fluc control or EOF1 1 day after adult eclosion and allowed to feed on blood 4 days post dsRNA microinjection, as shown in Fig. 1. Ovaries were dissected from dsRNA-injected mosquitoes 4 days PBM, and mature primary follicles were separated from dissected ovaries and thoroughly washed in 1 × PBS to completely remove shed follicular epithelial cells, secondary follicles, the germarium, and the ovarian tissues including common and lateral oviducts. Primary follicles were homogenized using a dounce homogenizer with pestle B, and the extracellular eggshells were filtered through a nylon mesh strainer (40 µm) and washed thoroughly with distilled water to remove oocyte cytosolic and membrane contents. Next, the enriched eggshells were homogenized in 6.0 M guanidine hydrochloride using a dounce homogenizer with pestle A and incubated at 37 °C overnight. The eggshell proteins were subsequently precipitated with 100% ETOH overnight at − 20 °C and pelleted by a centrifugation (16,000* g* for 15 min at 4 °C). Finally, eggshell proteins were resuspended with a protein sample buffer, quantified, and denatured in boiling water.

### Protein sample preparation for LC–MS/MS mass spectrometry

*Statistical *Protein sample preparation for mass spectrometry was carried out at the Analytical and Biological Mass Spectrometry Facility at the University of Arizona. Eggshell protein samples were separated about 1.0 cm into resolving gel on precast 12% SDS-PAGE gels (Bio-Rad, Hercules, CA). The gels were fixed with 50% methanol and 10% glacial acetic acid for 30 min and stained with GelCode Blue Stain Reagent (Thermo Fisher Scientific, San Jose, CA) overnight. Excised gel bands were destained with 50/50 methanol/50 mM ammonium bicarbonate. Disulfide bonds were reduced with dithiothreitol (25 mM) for 45 min at 60 °C, and free cysteines were alkylated with iodoacetamide (55 mM) at room temperature for 30 min. After washing with 50 mM ammonium 5 times with agitation for 10 min each, the bands were cut into small pieces and incubated with trypsin/lysC (Promega) digestion solution in 50 mM ammonium bicarbonate containing 0.1% ProteaseMAX (Promega) overnight at 37 °C. Peptides were extracted from the gels with 45% acetonitrile / 5% isopropyl alcohol / 0.2% formic acid by sonication for 10 min. The extraction was repeated once; supernatants were pooled and dried down in a speed vac. LC–MS/MS analysis was performed on a Q Exactive Plus mass spectrometer (Thermo Fisher Scientific) equipped with an EASY-Spray nanoESI source at the University of Arizona Analytical and Biological Mass Spectrometry Facility. Only proteins with at least six matching peptide hits were included as eggshell proteins and subjected to protein BLAST searches against the NCBI non-redundant protein database to determine their putative protein functions. The mass spectrometry proteomics data have been deposited to the ProteomeXchange Consortium via the PRIDE [48] partner repository with the dataset identifier PXD045606 and 10.6019/PXD045606.

### Statistical analysis

Statistical analyses were performed using GraphPad Prism Software (version 5.0; GraphPad, La Jolla, CA). Statistical significance for fecundity, melanization, viability, and RNAi knockdown efficiency was analyzed using unpaired Student’s *t* test and analysis of variance followed by Tukeys post hoc test. *P* values of < 0.05 were considered significantly different. All experiments were performed using at least three independent biological cohorts except the proteomics studies (two independent biological replicates).

### Supplementary Information

**Additional file 1:** **Table S1.** RNAi screening of *Aedes aegypti e*ggshell proteins.

**Additional file 2:** **Table S2.** Primers used for RNAi screening.

**Additional file 3:** **Video S1.** Time-lapse videos on a melanization process of eggs deposited by female *Aedes aegypti* mosquitoes that were microinjected with different dsRNAs. The photo images were taken by Canon EOS 700D/Rebel T5i. The videos are from dsRNA-Fluc control (top left), dsRNA- closca (top right), dsRNA- Nasrat (bottom left), and dsRNA-Nudel (bottom right). An oviposition paper was soaked with water periodically to prevent dryness.

**Additional file 4:** **Table S3.** Reproductive phenotypes associated with RNAi in* Aedes aegypti.*

**Additional file 5:** **Table S4.** Gene-specific primers used for RNAi and qPCR in *Aedes aegypti.*

**Additional file 6: Table S5.** Reproductive phenotypes associated with RNAi in* Aedes aegypti*

**Additional file 7:** **Table S6.** Reproductive phenotypes associated with RNAi in two gonotrophic cycles.

**Additional file 8:** **Table S7.** An in vitro follicle melanization assay in *Aedes aegypti*.

**Additional file 9: Table S8. **An in vitro follicle melanization assay using a Rhodamine B.

**Additional file 10:** **Table S9.** Reproductive phenotypes associated with RNAi in* Aedes aegypti.*

**Additional file 11:** **Table S10.** *Aedes aegypti* eggshell proteomic analyses. Data are available via ProteomeXchange with identifier PXD045606. The data was also deposited in the University of Arizona Research Data Repository (https://doi.org/10.25422/azu.data.24162528).

**Additional file 12.** Submission experimental data. Data table with all the experimental data excluding proteomics results.

## Data Availability

All data analyzed for this study are included in this published article, its supplementary files, and publicly available repositories. Proteomics raw data are available via ProteomeXchange with identifier PXD045606. The data was also deposited in the University of Arizona Research Data Repository (https://doi.org/10.25422/azu.data.24162528).

## Abbreviations

*A.*: *aegypti Aedes aegypti*
*A.*: *albopictus Aedes albopictus*
ACC: Acetyl CoA carboxylase
CATL3: Cysteine protease cathepsin L-like 3
cDNA: Complementary DNA
CT: Central tubercles
DCE: Dopachrome-converting enzymes
*D.*: *melanogaster Drosophila melanogaster*
dsRNA: Double-stranded RNA
EMS: Electron Microscopy Sciences
EN: Exochorionic network
EOF1: Eggshell organizing factor 1
ETOH: Ethanol
Fluc: Firefly luciferase
HMDS: Hexamethyldisilazane
LC–MS/MS: Liquid chromatography tandem mass spectrometry
mRNA: Messenger RNA
OCA: Outer chorionic area
PBM: Post blood meal
PBS: Phosphate-buffered saline
PCR: Polymerase chain reaction
PI: Protease inhibitor
PIPES: Piperazine-N, N′-bis(2-ethanesulfonic acid)
PMSF: Phenylmethylsulfonyl fluoride
PT: Peripheral tubercles
qPCR: Quantitative real-time PCR
RNAi: RNA interference
SEM: Scanning electron microscopy
SEM: Standard error of mean
