# Supplementary material for: Characterization of essential eggshell proteins from Aedes aegypti mosquitoes
Source: BMC Biol. 2023 Oct 13;21:214. doi: 10.1186/s12915-023-01721-z (PMC10576393; doi:10.1186/s12915-023-01721-z)
Supplement: Supplementary file 1 — Additional file 1: Table S1. RNAi screening of Aedes aegypti eggshell proteins. [file 12915_2023_1721_MOESM1_ESM.pdf]

Additional file 1.

Table S1. RNAi screening of *Aedes aegypti e*ggshell proteins.

Vectorbase ID	GenBank ID	Putative functions	*RNAi phenotypic effects* Eggs	Hatching
AAEL000361	EAT48607	Trypsin inhibitor-like/serpin	NO	NO
AAEL000363	EAT48611	Trypsin inhibitor-like/serpin	NO	NO
AAEL000375	EAT48605	Trypsin inhibitor-like/serpin	NO	NO
AAEL000507	EAT48446	Chorion peroxidase, CP4	NO	NO
AAEL000961	EAT47957	Closca	YES	YES
AAEL002196	EAT46597	Cysteine proteinase L-like, CATL3	YES	YES
AAEL002382	EAT46452	Unknown	NO	NO
AAEL003110	EAT45649	Chitinase domain	NO	NO
AAEL004202	EAT44412	Unknown	NO	NO
AAEL004386	EAT44219	Chorion peroxidase, CP1	NO	NO
AAEL004390	EAT44218	Chorion peroxidase, CP2	NO	NO
AAEL004401	EAT44216	Chorion peroxidase, CP7	NO	NO
AAEL005098	EAT43477	Trypsin inhibitor-like/serpin	NO	NO
AAEL005648	EAT42848	Clip-domain serine protease	NO	NO
AAEL005861	EAT42645	Vacuolar sorting protein	NO	NO
AAEL006830	EAT41553	Dopachrome converting enzyme, DCE2	YES	YES
AAEL006985	EAT41324	Dopachrome converting enzyme, DCE3	NO	NO
AAEL007096	EAT41240	Dopachrome converting enzyme, DCE4	YES	NO
AAEL007415	EAT40867	Laccase-like multicopper oxidases	NO	NO
AAEL007641	EAT40646	Transglutaminase	NO	NO
AAEL008829	EAT39370	Nasrat	YES	YES
AAEL009290	EAT38853	Unknown	NO	NO
AAEL009452	EAT38674	Unknown	NO	NO
AAEL009746	EAT38349	Unknown	NO	NO
AAEL010544	EAT37465	Unknown	NO	NO
AAEL010848	EAT37145	Dopachrome converting enzyme, DCE5	YES	NO
AAEL011238	EAT36701	Trypsin inhibitor-like/serpin	NO	NO
AAEL012586	EAT35235	Unknown	NO	NO
AAEL013027	EAT34764	Vitelline membrane protein, 15a1	NO	NO
AAEL013936	EAT33799	Trypsin inhibitor-like/serpin	NO	NO
AAEL014561	EAT33176	Vitelline membrane protein, 15a3	NO	NO
AAEL015203	EAT32616	Unknown	NO	NO
AAEL017403	EJY58008	Vitelline membrane protein, 15a2	NO	NO
AAEL017467	EJY57339	Chorion peroxidase, CP6	NO	NO
