# Supplementary material for: Characterization of essential eggshell proteins from Aedes aegypti mosquitoes
Source: BMC Biol. 2023 Oct 13;21:214. doi: 10.1186/s12915-023-01721-z (PMC10576393; doi:10.1186/s12915-023-01721-z)
Supplement: Supplementary file 2 — Additional file 2: Table S2. Primers used for RNAi screening. [file 12915_2023_1721_MOESM2_ESM.pdf]

Additional file 2.

Table S2. Primers used for RNAi screening.

Vectorbase ID	Gene-specific RNAi primers	(5' - 3')	Vectorbase ID	Gene-specific RNAi primers	(5' - 3')
AAEL000361	Forward	GTAGCGATTGTTGTTCTAGCG	AAEL007415	Forward	TCAGTGCCGACCAGCAAGT
	Reverse	GGCGTAAGTAACTTGCACACGG		Reverse	CTGAGATTGTCTTGTTGGACTTC
AAEL000363	Forward	CCCTGTGCCGACCCAAACGA	AAEL007641	Forward	CGCTCGCAACGTGCATTGG
	Reverse	CGGTGTGGTCGTAGTAATACA		Reverse	GGACACCACGCACGCTGCAA
AAEL000375	Forward	ATGCAGCTTCCAATATGTGCTAT	AAEL008829	Forward	GAGCCCATTCAGAACCTCCT
	Reverse	GCGGCTTCGGCGTAGGCTT		Reverse	AGCGTAACTCCGTTGACGTA
AAEL000507	Forward	TACAGCTCTGCGTGCATCTG	AAEL009290	Forward	ATCGAGGGATTGATGGAAGG
	Reverse	CCACAATCGGTCTTCGTCAG		Reverse	CCGTCCGAGTAGTGGATCGC
AAEL000961	Forward	GGCAAGGGCTTCTACAACGT	AAEL009452	Forward	GTCCTCCATCTCTTTGGTGA
	Reverse	CCGTTCAAAGTATGCTCCAC		Reverse	TCACCAACCAGCTCTTCTCG
AAEL002196	Forward	TGAAGAAACAACTGCTGTGG	AAEL009746	Forward	CAGCCTACATCGTTGACCTA
	Reverse	CCATCTGCTGGGTACTGAC		Reverse	TGTCGAAGCACAACCGATGGTT
AAEL002382	Forward	GCCCATGTGAACTTCCCTTGCC	AAEL010544	Forward	CAGCGGGATCAGAACCAGGAT
	Reverse	GATACCTCGCCCTGTTGAAC		Reverse	CATAAGATCCGTCAGACCGTC
AAEL003110	Forward	TCCAACCAGGAGTCGAGTGA	AAEL010848	Forward	GCCCTCGACTCAGGCATTTG
	Reverse	CGCCAATTCCACCGAGTTG		Reverse	TGCGTGCTCAAGCGACACTC
AAEL004202	Forward	TCCTACGGCGAAGCTGGTTC	AAEL011238	Forward	CAACCAGTTGATGGCAGGATAC
	Reverse	GACTCTCGTTTGTCTGCTTC		Reverse	CCGTTGTGCTTCACATAACC
AAEL004386	Forward	TGAGGGAACACAACCGACTA	AAEL012586	Forward	GCCGACAGGGACCGATGATG
	Reverse	TAAACCTGTGCCAAGAGTGC		Reverse	GCCGAAATGTTGATCTTGTGTAC
AAEL004390	Forward	TGAGGGAACACAACCGACTA	AAEL013027	Forward	TTCCCATCCAACTCAGTAACCAT
	Reverse	TAAACTCGCGCCAGAAGAGC		Reverse	TTCCGCTGCATCTTCAAGAG
AAEL004401	Forward	CCACACTGGTCTGACGACAT	AAEL013936	Forward	TTAGCAATAGTTTCTCACTGCCA
	Reverse	CGCCTACGTAAAGATCGACGT		Reverse	GCCTGTGGGCTTCGATTGG
AAEL005098	Forward	ATGAAGTTGGCAATCATTTGTGT	AAEL014561	Forward	CGGAAGGAATCCATCCAACTT
	Reverse	GCTCCAGGACAATCGCACAG		Reverse	CAGTCCAATCGATGATCCGC
AAEL005648	Forward	GCCAAAGCCGATAGCCATC	AAEL015203	Forward	GTGTTGGTGCCGAAGAAGAG
	Reverse	CTAGGCATGTTGAGAGCACC		Reverse	TAGCACTTCAACTCGGATGACTT
AAEL005861	Forward	TGTGATGGCGATGACGACTG	AAEL017403	Forward	CCAGCGTGGTACAACAGTAAATC
	Reverse	CTCATCACTTCCATCCTTGCA		Reverse	CCGTTCCTTGGTCCTGGTTC
AAEL006830	Forward	TGTGGAAATCGTCGGTGGT	AAEL017467	Forward	TACAGCTCTGCGTGCATCTG
	Reverse	TGTAGGCGAAGGTGTCCTC		Reverse	CCACAATCGGTCTTCGTCAG
AAEL006985	Forward	CTCCCGGTTGGAATCGAAAG	AAEL012336	Forward	AGCCCGTCCAAGAGGAAGTT
	Reverse	GTCCGTAGTCCAGTTCATTGG	(EOF1)	Reverse	CTCGGATGGTACTCACACAA
AAEL007096	Forward	GCAAGAAGTGGCGACAAGAC	U47295	Forward	AGCACTCTGATTGACAAATACGA
	Reverse	CGTCCACCCAGATAGGTGAA	(Luciferase)	Reverse	AGTTCACCGGCGTCATCGTC

T7 promoter sequence (5' TAATACGACTCACTATAGGGAGA 3') was added in 5' of each RNAi primer.
