# Supplementary material for: Characterization of essential eggshell proteins from Aedes aegypti mosquitoes
Source: BMC Biol. 2023 Oct 13;21:214. doi: 10.1186/s12915-023-01721-z (PMC10576393; doi:10.1186/s12915-023-01721-z)
Supplement: Supplementary file 4 — Additional file 4: Table S3. Reproductive phenotypes associated with RNAi in Aedes aegypti. [file 12915_2023_1721_MOESM4_ESM.pdf]

Additional file 4.

Table S3. Reproductive phenotypes associated with RNAi in *Aedes aegypti.*

RNAi	Fluc	Nasrat	Closca	Polehole	Nudel
Fecundity					
Number of mosquitoes examined	24	26	25	27	24
Total number of eggs oviposited	2273	2128	2073	2359	1955
Mean number of eggs oviposited	94.7	81.8	82.9	87.4	81.5
Eggshell melanization					
Number of eggs examined	2273	2128	2073	2359	1955
Incompletely tanned eggs oviposited	23	1520	1749	1955	1938
Incomplete eggshell melanization (%)	1.01%	79.89%	84.37%	82.87%	99.13%
Egg viability					
Number of eggs examined	666	2128	2073	2359	1955
Number of eggs hatched	626	231	184	226	5
Egg viability (%)	93.99%	10.86%	8.88%	9.58%	0.26%

Egg phenotypes are shown in Figure 2.

dsRNA was microinjected 4 days prior to blood feeding as shown in Figure 1.
