# Supplementary material for: Characterization of essential eggshell proteins from Aedes aegypti mosquitoes
Source: BMC Biol. 2023 Oct 13;21:214. doi: 10.1186/s12915-023-01721-z (PMC10576393; doi:10.1186/s12915-023-01721-z)
Supplement: Supplementary file 5 — Additional file 5: Table S4. Gene-specific primers used for RNAi and qPCR in Aedes aegypti. [file 12915_2023_1721_MOESM5_ESM.pdf]

Additional file 5.

Table S4. Gene-specific primers used for RNAi and qPCR in *Aedes aegypti.*

Genes		Primer sequence (5' to 3')
Gene-specific primers used for RNAi		
Polehole, AAEL022628	Forward	TGTACGGAAGGCAGGATTC
	Reverse	GGGTAGAGTTTGGTCAGGTT
Nudel, AAEL016971	Forward	GCAAGCTGATGAAGTTCCACA
	Reverse	CCACGTCTTGTTCTCGGTTTC
Gene-specific primers used for qPCR		
Nasrat	Forward	CTGAACACCGATCAGACGAT
	Reverse	TGAGCGTATTCTTGCGTTCGTA
Closca	Forward	GCCACCGACGTGCTGTTGAA
	Reverse	TCCCGTAGTTTAGCGTAGTTC
Polehole	Forward	ATAGTTCATCAGTTTCGATGCC
	Reverse	AACATCACATCGAACAAGAGTGT
Nudel	Forward	CACTTCGAGAACCAACATAAGG
	Reverse	GGAAGGTGATGTGCGTTAG
DCE2	Forward	GCTAACATTGCCATCGACATG
	Reverse	GCCAGGACTTGTTCTTCTCAA
DCE4	Forward	TGTAGATTCCGCGACGTTCT
	Reverse	CGTAGGTGATCCGTAGCAAT
DCE5	Forward	TGGAACACTGATCAACCGTACAA
	Reverse	GACAATGAGTACATCAGAGCATC
CATL3	Forward	GCCCTCAATGGACAGATTATG
	Reverse	GATCCTCCAGCACATCCCTT
Ribsomal protein S7	Forward	ACCGCCGTCTACGATGCCA
	Reverse	ATGGTGGTCTGCTGGTTCTT

T7 promoter sequence (5' TAATACGACTCACTATAGGGAGA 3') was added in 5' of each RNAi primer.
