# Supplementary material for: Characterization of essential eggshell proteins from Aedes aegypti mosquitoes
Source: BMC Biol. 2023 Oct 13;21:214. doi: 10.1186/s12915-023-01721-z (PMC10576393; doi:10.1186/s12915-023-01721-z)
Supplement: Supplementary file 6 — Additional file 6: Table S5. Reproductive phenotypes associated with RNAi in Aedes aegypti [file 12915_2023_1721_MOESM6_ESM.pdf]

Additional file 6.

Table S5. Reproductive phenotypes associated with RNAi in *Aedes aegypti*

RNAi	Fluc	Nasrat	Closca	Polehole	Nudel
Fecundity					
Number of mosquitoes examined	12	12	12	12	12
Total number of eggs oviposited	1076	1012	1022	1024	976
Mean number of eggs oviposited	89.7	84.3	85.2	85.3	81.3
Eggshell melanization					
Number of eggs examined	1076	1012	1022	1024	976
Incompletely tanned eggs oviposited	14	30	22	31	964
Incomplete eggshell melanization (%)	1.30%	2.96%	2.15%	3.03%	98.77%
Egg viability					
Number of eggs examined	364	368	348	342	976
Number of eggs hatched	333	332	312	302	8
Egg viability (%)	91.48%	90.22%	89.66%	88.30%	0.82%

Egg phenotypes are shown in Fig. 4.

dsRNA was microinjected immediately after blood feeding as shown in Fig. 4.
