# Supplementary material for: Characterization of essential eggshell proteins from Aedes aegypti mosquitoes
Source: BMC Biol. 2023 Oct 13;21:214. doi: 10.1186/s12915-023-01721-z (PMC10576393; doi:10.1186/s12915-023-01721-z)
Supplement: Supplementary file 7 — Additional file 7: Table S6. Reproductive phenotypes associated with RNAi in two gonotrophic cycles. [file 12915_2023_1721_MOESM7_ESM.pdf]

Additional file 7.

Table S6. Reproductive phenotypes associated with RNAi in two gonotrophic cycles.

	First gonotrophic cycle		Second gonotrophic cycle	
RNAi	Fluc	Nudel	Fluc	Nudel
Fecundity				
Number of mosquitoes examined	12	12	12	12
Total number of eggs oviposited	1007	902	807	731
Mean number of eggs oviposited	83.9	75.2	67.3	60.9
Eggshell melanization				
Number of eggs examined	1007	902	807	731
Incompletely tanned eggs oviposited	11	890	22	21
Incomplete eggshell melanization (%)	1.09%	98.67%	2.73%	2.87%
Egg viability				
Number of eggs examined	365	902	383	393
Number of eggs hatched	335	7	338	337
Egg viability (%)	91.78%	0.78%	88.25%	85.75%

Egg phenotypes are shown in Fig. 5.

dsRNA was microinjected immediately after blood feeding as shown in Fig. 5.
