# Supplementary material for: Characterization of essential eggshell proteins from Aedes aegypti mosquitoes
Source: BMC Biol. 2023 Oct 13;21:214. doi: 10.1186/s12915-023-01721-z (PMC10576393; doi:10.1186/s12915-023-01721-z)
Supplement: Supplementary file 8 — Additional file 8: Table S7. An in vitro follicle melanization assay in Aedes aegypti. [file 12915_2023_1721_MOESM8_ESM.pdf]

Additional file 8.

Table S7. An *in vitro* follicle melanization assay in *Aedes aegypti*.

RNAi treatment	Fluc	Nasrat	Closca	Polehole	Nudel
Number of mosquitoes examined	5	5	5	5	5
Total number of follicles examined	118	121	105	107	113
Total number of follicles melanized	114	6	13	15	4
Follicle melanized (%)	96.6%	5.0%	12.4%	14.0%	3.5%

Protease inhibitor (PI) treatment on wildtype mosquitoes		Inhibitors were added after follicle dissection (min)			
		PI			PMSF
	Untreated	0	10	20	0
Number of mosquitoes examined	5	5	5	5	5
Total number of follicles examined	122	146	130	132	129
Total number of follicles melanized	118	3	125	126	115
Follicle melanized (%)	96.7%	2.1%	96.2%	95.5%	89.1%

Follicle phenotypes are shown in Figure 6.
dsRNA was microinjected 4 days prior to blood feeding as shown in Figure 1.
