# Supplementary material for: Characterization of essential eggshell proteins from Aedes aegypti mosquitoes
Source: BMC Biol. 2023 Oct 13;21:214. doi: 10.1186/s12915-023-01721-z (PMC10576393; doi:10.1186/s12915-023-01721-z)
Supplement: Supplementary file 9 — Additional file 9: Table S8. An in vitro follicle melanization assay using a Rhodamine B. [file 12915_2023_1721_MOESM9_ESM.pdf]

Additional file 9.

Table S8. An *in vitro* follicle melanization assay using a Rhodamine B.

RNAi treatment	Fluc	Nasrat	Closca	Polehole	Nudel
Number of mosquitoes examined	5	5	5	5	5
Total number of follicles examined	127	133	123	123	117
Total number of follicles stained	3	126	119	118	113
Follicles stained (%)	2.4%	94.7%	96.7%	95.9%	96.6%

Follicle stained phenotypes are shown in Figure 6.

dsRNA was microinjected 4 days prior to blood feeding as shown in Figure 1.
