# Supplementary material for: Characterization of essential eggshell proteins from Aedes aegypti mosquitoes
Source: BMC Biol. 2023 Oct 13;21:214. doi: 10.1186/s12915-023-01721-z (PMC10576393; doi:10.1186/s12915-023-01721-z)
Supplement: Supplementary file 10 — Additional file 10: Table S9. Reproductive phenotypes associated with RNAi in Aedes aegypti. [file 12915_2023_1721_MOESM10_ESM.pdf]

Additional file 10.

Table S9. Reproductive phenotypes associated with RNAi in *Aedes aegypti.*

RNAi	Fluc	DCE2	DCE4	DCE5	CATL3
Fecundity					
Number of mosquitoes examined	23	24	25	25	26
Total number of eggs oviposited	2022	2128	2144	2130	2195
Mean number of eggs oviposited	87.9	88.7	85.8	85.2	84.4
Eggshell melanization					
Number of eggs examined	2022	2128	2144	2130	2195
Incompletely tanned eggs oviposited	19	2036	27	36	952
Incomplete eggshell melanization (%)	0.94%	95.68%	1.26%	1.69%	43.37%
Egg viability					
Number of eggs examined	605	751	685	714	767
Number of eggs hatched	557	45	623	630	33
Egg viability (%)	92.07%	5.99%	90.95%	88.24%	4.30%

Egg phenotypes are shown in Figure 7.

dsRNA was microinjected 4 days prior to blood feeding as shown in Figure 1.
