# Supplementary material for: Characterization of essential eggshell proteins from Aedes aegypti mosquitoes
Source: BMC Biol. 2023 Oct 13;21:214. doi: 10.1186/s12915-023-01721-z (PMC10576393; doi:10.1186/s12915-023-01721-z)
Supplement: Supplementary file 11 — Additional file 11: Table S10. Aedes aegypti eggshell proteomic analyses. Data are available via ProteomeXchange with identifier PXD045606. The data was also deposited in the University of Arizona Research Data Repository (https://doi.org/10.25422/azu.data.24162528). [file 12915_2023_1721_MOESM11_ESM.pdf]

Additional file 11.

Table S10. Raw data on eggshell proteomic analyses in *Aedes aegypti*.

Vectorbase ID	GenBank ID	Putative functions	# peptide hits RNAi-Fluc	# peptide hits RNAi-EOF1
AAEL010434	AAA18221	Vitellogenin	244	323
AAEL006126	EAT42292	Vitellogenin	281	222
AAEL006138	XP_001657509	Vitellogenin	278	219
AAEL006830	XP_001658066	Dopachrome converting enzyme	180	230
AAEL004390	XP_001649029	Chorion peroxidase	153	170
AAEL010872	XP_001661124	Odorant binding protein	172	141
AAEL026038	XP_021692994	Chorion peroxidase	160	146
AAEL000961	XP_021708553	Closca	143	161
AAEL013492	EAT34242	Phenoloxidase	156	147
AAEL008829	XP_001659577	Nasrat	112	128
AAEL007415	EAT40867	Multicopper oxidase	87	106
AAEL004386	P82600	Chorion peroxidase	100	82
AAEL011763	XP_001661890	Phenoloxidase	93	78
AAEL022628	XP_021707815	Polehole	67	88
AAEL003110	EAT45649	Chitinase	86	61
AAEL003511	XP_001656921	Odorant binding protein	108	33
AAEL009433	XP_001649879	Odorant binding protein	95	42
AAEL009599	XP_001660278	Odorant binding protein	98	37
AAEL022918	XP_021710656	Glucose dehydrogenase	65	61
AAEL006396	EAT42032	Odorant binding protein	90	35
AAEL002851	ABH03477	Tubulin beta	56	63
AAEL026563	XP_021695347	Odorant binding protein	66	42
AAEL005752	XP_001651411	Lysosomal alpha-mannosidase	61	43
AAEL006563	P42660	Vitellogenic carboxypeptidase	42	55
AAEL006393	EAT42030	Odorant binding protein	74	23
AAEL008797	XP_001659527	Titin	61	33
AAEL005325	EAT43230	Dopachrome converting enzyme	53	40
AAEL006642	XP_001652144	Tubulin alpha-1	43	47
AAEL000796	EAT48134	Odorant binding protein	55	31
AAEL006398	EAT42033	Odorant binding protein	57	28
AAEL005198	EAT43356	Juvenile hormone esterase	49	35
AAEL016971	EJY57924	Nudel	54	26
AAEL009642	XP_001653891	Cathepsin B-like cysteine proteinase 3	57	23
AAEL007096	XP_001658111	Dopachrome converting enzyme	42	35
AAEL013338	XP_001663495	Lethal(2)essential for life	43	31
AAEL017467	EJY57339	Chorion peroxidase	48	25
AAEL011758	ABF18058	Cyclophylin	42	27
AAEL011197	AAY81972	Actin	34	31
AAEL011116	XP_001655111	14-3-3 protein epsilon	32	27
AAEL019403	ABF18332	Heat shock 70	40	14
AAEL022697	XP_021706511	Odorant binding protein	40	14
AAEL007339	XP_001652677	Heat shock protein 67b2	32	22
AAEL006328	XP_021710623	Chitinase	38	14
AAEL000889	XP_001651634	Carboxylic ester hydrolase	34	18
AAEL003315	EAT45429	Odorant binding protein	30	21
AAEL005733	XP_021701099	Myosin heavy chain	12	38
AAEL009895	EAT38188	Neprilysin	36	14
AAEL017096	XP_011493435	Elongation factor 1-alpha	30	19
AAEL000144	EAT48853	Chitinase	39	10
AAEL000377	EAT48658	Odorant binding protein	30	18
AAEL015116	AAG02219	Prophenoloxidase	15	31
AAEL012062	XP_021693479	Na+/K+ ATPase alpha subunit	14	32
AAEL013719	EAT34018	Odorant binding protein	29	16
AAEL015289	EAT32570	Uncharacterized protein	18	25
AAEL004434	XP_001649141	Transketolase-like protein 2	18	24

Continued on next page

Table S10. Raw data on eggshell proteomic analyses in *Aedes aegypti* (continued).

Vectorbase ID	GenBank ID	Putative functions	# peptide hits RNAi-Fluc	# peptide hits RNAi-EOF1
AAEL010848	XP_021698010	Dopachrome converting enzyme	32	10
AAEL013501	EAT34239	Prophenoloxidase	20	22
AAEL000507	EAT48446	Chorion peroxidase	33	9
AAEL017501	EJY57337	NA-vitelline membrane	17	24
AAEL011708	EAT36186	Heat shock protein 83	28	13
AAEL014876	EAT32887	Odorant binding protein	28	12
AAEL003393	EAT45330	ATP synthase beta subunit	26	13
AAEL022484	XP_021706407	Odorant binding protein	25	14
AAEL000318	EAT48660	Odorant binding protein	20	19
AAEL000833	XP_001651310	Odorant binding protein	23	16
AAEL015312	EAT32556	Cysteine proteinase-1	22	16
AAEL004172	EAT44445	Alpha-Tubulin	16	22
AAEL014431	EAT33288	Odorant binding protein	22	15
AAEL004028	XP_001648297	Glucose dehydrogenase	25	12
AAEL001174	EAT47749	Odorant binding protein	28	8
AAEL009955	XP_021707150	Apolipophorins	6	29
AAEL000344	XP_001655549	Odorant binding protein	20	15
AAEL009038	XP_021697410	Serine protease/prolylcarboxypeptidase	23	12
AAEL007599	EAT40702	Cathepsin B	27	7
AAEL002023	EAT46826	Chitinase	19	14
AAEL014430	EAT33287	Odorant binding protein	20	11
AAEL006820	XP_001658058	Lipid storage droplets-binding protein 2	24	7
AAEL011300	EAT36638	Uncharacterized protein	17	14
AAEL004500	AAK01430	Eukaryotic translation elongation	17	13
AAEL006336	EAT42103	Chitinase	28	2
AAEL008787	O16109	V-ATPase subunit A	15	15
AAEL014222	EAT33503	Vitellogenin receptor	17	12
AAEL024387	XP_021712253	Serine protease	19	10
AAEL018219	XP_021705132	BM-specific heparan sulfate proteoglycan	11	17
AAEL005766	XP_001651423	Fructose-bisphosphate aldolase	21	7
AAEL001593	EAT47332	GAPDH 1	14	13
AAEL006271	EAT42157	Superoxide dismutase 3	15	11
AAEL005756	EAT42731	Uncharacterized protein	20	6
AAEL012175	XP_001655906	ATP synthase alpha subunit	16	9
AAEL001061	XP_011493351	Glutathione S transferase	14	11
AAEL005759	EAT42734	Uncharacterized protein	21	4
AAEL013359	XP_001656676	DEAD box ATP-dependent RNA helicase	11	14
AAEL000496	XP_001656955	Chorion peroxidase	15	10
AAEL006322	EAT42110	Odorant binding protein	11	14
AAEL001189	EAT47748	Uncharacterized protein	13	11
AAEL010912	EAT37053	DPPIV-SP, Omega	12	12
AAEL013279	XP_001663442	Peptidyl-prolyl cis-trans isomerase	14	9
AAEL017502	EJY57472	Uncharacterized protein	20	3
AAEL010874	EAT37093	Odorant binding protein	20	2
AAEL007097	XP_001658126	4-nitrophenylphosphatase	15	7
AAEL022038	XP_021695232	Uncharacterized protein	15	6
AAEL000703	XP_001650265	Glycogen phosphorylase	17	4
AAEL003513	EAT45173	Uncharacterized protein	16	4
AAEL000179	XP_001658903	Ubiquitin-conjugating enzyme E2	11	9
AAEL027038	XP_021710733	Uncharacterized protein	11	9
AAEL008799	XP_001659528	Uncharacterized protein	15	5
AAEL010097	XP_001654240	Maternal protein exuperantia	12	7
AAEL023983	ABF18051	40S ribosomal protein S4	9	10
AAEL012054	EAT35810	Uncharacterized protein	11	8
AAEL013353	XP_001656670	Chicadee/profilin	7	12

Continued on next page

Table S10. Raw data on eggshell proteomic analyses in *Aedes aegypti* (continued).

Vectorbase ID	GenBank ID	Putative functions	# peptide hits	
			RNAi-Fluc	RNAi-EOF1
AAEL002160	XP_001654823	GTP-binding protein	11	8
AAEL003094	EAT45648	Chitinase	15	4
AAEL026426	XP_021695306	Vitelline membrane protein-like	10	8
AAEL009207	EAT38962	Uncharacterized protein	13	5
AAEL02734	XP_021694355	Uncharacterized protein	11	7
AAEL012960	XP_001663145	Pendulin (NLS-receptor)	14	4
AAEL007432	EAT40884	Serine protease	18	0
AAEL008862	XP_001653480	Metalloprotease	14	3
AAEL001863	AAT36732	Zinc carboxypeptidase	12	5
AAEL001153	EAT47747	Uncharacterized protein	14	3
AAEL005216	EAT43331	Uncharacterized protein	14	3
AAEL001588	XP_001653563	Glutamate carboxypeptidase	12	5
AAEL011808	EAT36073	Gglucose dehydrogenase	11	6
AAEL008640	EAT39566	Odorant binding protein	13	4
AAEL016984	XP_011493026	Glyceraldehyde-3-phosphate dehydrogenase	11	5
AAEL004388	EAT44220	Chorion peroxidase	0	16
AAEL004516	XP_001649345	Odorant binding protein	9	7
AAEL015288	EAT32569	Uncharacterized protein	8	8
AAEL006885	XP_001652301	14-3-3 zeta	9	7
AAEL019604	XP_021698211	Uncharacterized protein	10	5
AAEL010585	XP_001654680	TER94	15	0
AAEL010821	XP_001655016	60S ribosomal protein LP0	8	7
AAEL012996	XP_021709703	Rho guanine dissociation factor	10	5
AAEL008500	XP_001659287	DEAD box ATP-dependent RNA helicase	11	3
AAEL001965	ABF18180	Chitinase/imaginal disc growth factor	5	9
AAEL005901	XP_001663344	40S ribosomal protein S3a	8	6
AAEL020238	Q16ZR8	40S ribosomal protein SA	10	4
AAEL001845	EAT47019	Sepiapterin reductase	8	6
AAEL003525	EAT45172	Odorant binding protein	14	0
AAEL005422	XP_001650875	Pyrroline-5-carboxylate dehydrogenase	11	3
AAEL004984	XP_001650136	Cullin-associated NEDD8-dissociated protein	14	0
AAEL000827	EAT48139	Odorant binding protein	10	4
NA	CAF02084	Odorant binding protein	12	2
AAEL003872	XP_001664282	Translationally-controlled tumor protein	8	6
AAEL001179	EAT47746	Odorant binding protein	10	4
AAEL009994	XP_001660544	60S ribosomal protein L4	9	4
AAEL012897	XP_001663037	Aconitase, mitochondrial	10	3
AAEL009496	XP_001660169	40S ribosomal protein S7	6	7
AAEL014548	EAT33191	Thioredoxin peroxidase	6	7
AAEL019408	AAL37254	2-Cys thioredoxin peroxidase	9	4
AAEL028058	XP_021695267	Odorant binding protein	9	4
AAEL012035	XP_001655825	Vacuolar ATP synthase subunit e	7	6
AAEL014274	XP_001648333	Uncharacterized protein	7	6
AAEL000821	EAT48127	Odorant binding protein	11	2
AAEL017349	XP_011493320	HSP70	8	4
AAEL019579	XP_021704200	Furin-like protease 2	7	5
AAEL001432	EAT47483	Protein disulfide isomerase	9	3
AAEL009097	EAT39077	Cathepsin	7	5
AAEL004978	XP_001650127	DEAD box ATP-dependent RNA helicase	9	3
AAEL003820	EAT44826	Histone H2A-like	6	6
AAEL000758	EAT48168	Ubiquitin activating enzyme 1	11	1
AAEL017451	XP_011493087	Angiotensin converting enzyme	9	3
AAEL009077	EAT39089	Alkaline phosphatase	9	3
AAEL008280	EAT39971	Uncharacterized protein	5	6
AAEL010168	XP_001654299	40S ribosomal protein S2	6	5

Continued on next page

Table S10. Raw data on eggshell proteomic analyses in *Aedes aegypti* (continued).

Vectorbase ID	GenBank ID	Putative functions	# peptide hits RNAi-Fluc	# peptide hits RNAi-EOF1
AAEL001495	EAT47408	Uncharacterized protein	2	9
AAEL019935	XP_021709761	Midline fasciclin	7	4
AAEL000837	EAT48137	Odorant binding protein	7	4
AAEL008481	XP_001659268	60S ribosomal protein L18	6	5
AAEL000846	XP_001651309	Odorant binding protein	13	8
AAEL011870	XP_001662014	Trailer hitch,protein LSM14 homolog B	9	2
AAEL010919	EAT37047	Prophenoloxidase	5	6
AAEL007003	EAT41362	Odorant binding protein	7	4
AAEL008381	EAT39841	Peptide transporter	5	6
AAEL004532	EAT44083	Glyoxylate reductase	6	5
AAEL005798	XP_001651458	ATP synthase subunit beta vacuolar	6	5
AAEL002978	EAT45789	Aminopeptidase	8	3
AAEL012904	XP_001663045	Rab gdp-dissociation inhibitor	8	3
AAEL014719	EAT33023	Uncharacterized protein	7	4
AAEL007962	XP_001658750	Glutathione transferase	7	4
AAEL004755	EAT43826	Enoyl-CoA delta isomerase	10	1
AAEL014238	AAC31639	DOPA decarboxylase	9	1
AAEL011288	ABF18271	Eukaryotic translation elongation factor 1-γ	8	2
AAEL009287	XP_001659895	Ran, GTP-binding nuclear protein	6	4
AAEL006389	ABE72972	Cathepsin L	8	2
AAEL007915	Q170J7	Moesin	7	3
AAEL004856	XP_001649919	Odorant binding protein	8	2
AAEL001487	EAT47390	Odorant binding protein	7	3
AAEL006977	XP_001652452	Ser/thr protein phosphatase 2a reg. subunit a	8	2
AAEL009882	XP_001654079	Retinoblastoma-binding protein 4	6	4
AAEL011764	EAT36127	Phenoloxidase	2	8
AAEL003404	XP_001656805	Uncharacterized protein	0	10
AAEL022214	XP_021707151	γ-interferon inducible lys. thiol reductase	7	3
AAEL007236	ABF18366	Uncharacterized protein	6	4
AAEL000987	XP_001657711	60S ribosomal protein L8	6	3
AAEL012609	EAT35209	γ-aminobutyric acid transaminase	6	3
AAEL002542	XP_001655586	Triosephosphate isomerase	7	2
AAEL006836	XP_001652256	Dihydropteridine reductase	7	2
AAEL009080	XP_021695478	Importin 7	8	1
AAEL006670	EAT41719	Vitelline membrane protein 15a-3	8	0
AAEL010403	XP_001654538	Achaete scute target 1	7	1
AAEL000641	XP_011493116	Protein disulfide isomerase	6	2
AAEL022104	ABF18250	60S ribosomal protein L3	6	2
AAEL002861	ABF18383	Saccheropin dehydrogenase 1	7	1
AAEL001112	EAT47794	Ubiquitin specific protease 5	8	0
AAEL001605	XP_001659738	Mapmodulin/microtubule binding protein	2	6
AAEL013275	XP_001663434	Female sterile (2) ketel, importin beta	6	2
AAEL017315	EJY57568	HSC70	8	0
AAEL000109	XP_001657693	Enolase-phosphatase E1-like	6	2
AAEL013857	XP_001647837	Serine protease immune response integrator	1	6
AAEL012427	XP_001662561	Uncharacterized protein	7	0
AAEL005832	EAT42655	Programmed cell death 4	6	1
AAEL010506	XP_001660884	GTP-binding protein alpha subunit, gna	1	6
AAEL001035	EAT47891	Ca2+-binding protein Regucalcin/SMP30	6	1
AAEL009142	XP_001659779	Prolyl endopeptidase	6	0
AAEL013284	AAO43403	Uncharacterized protein	6	0
AAEL010698	EAT37289	Artemis	6	0
AAEL001401	XP_001659164	Leucine-rich immune protein	0	6
AAEL009746	EAT38349	Chitinase-domain	6	0
AAEL007014	EAT41361	Odorant binding protein	6	0
